# Autophagy exacerbates electrical remodeling in atrial fibrillation by ubiquitin-dependent degradation of L-type calcium channel

**DOI:** 10.1038/s41419-018-0860-y

**Published:** 2018-08-29

**Authors:** Yue Yuan, Jing Zhao, Yongtai Gong, Dingyu Wang, Xiaoyu Wang, Fengxiang Yun, Zhaorui Liu, Song Zhang, Wenpeng Li, Xinbo Zhao, Li Sun, Li Sheng, Zhenwei Pan, Yue Li

**Affiliations:** 1 Department of Cardiology, the First Affiliated Hospital, Harbin Medical University, 150001 Harbin, China; 2 Key Laboratory of Cardiac Diseases and Heart Failure, Harbin Medical University, 150001 Harbin, China; 3 Department of Pharmacology, Harbin Medical University, 150081 Harbin, China; 4 Institute of Metabolic Disease, Heilongjiang Academy of Medical Science, 150081 Harbin, China

## Abstract

Autophagy, a bidirectional degradative process extensively occurring in eukaryotes, has been revealed as a potential therapeutic target for several cardiovascular diseases. However, its role in atrial fibrillation (AF) remains largely unknown. This study aimed to determine the role of autophagy in atrial electrical remodeling under AF condition. Here, we reported that autophagic flux was markedly activated in atria of persistent AF patients and rabbit model of atrial rapid pacing (RAP). We also observed that the key autophagy-related gene7 (ATG7) significantly upregulated in AF patients as well as tachypacing rabbits. Moreover, lentivirus-mediated *ATG7* knockdown and overexpression in rabbits were employed to clarify the effects of autophagy on atrial electrophysiology via intracardiac operation and patch-clamp experiments. Lentivirus-mediated *ATG7* knockdown or autophagy inhibitor chloroquine (CQ) restored the shortened atrial effective refractory period (AERP) and alleviated the AF vulnerability caused by tachypacing in rabbits. Conversely, *ATG7* overexpression significantly promoted the incidence and persistence of AF and decreased L-type calcium channel (Cav1.2 α-subunits), along with abbreviated action potential duration (APD) and diminished L-type calcium current (*I*_Ca,L_). Furthermore, the co-localization and interaction of Cav1.2 with LC3B-positive autophagosomes enhanced when autophagy was activated in atrial myocytes. Tachypacing-induced autophagic degradation of Cav1.2 required ubiquitin signal through the recruitment of ubiquitin-binding proteins RFP2 and p62, which guided Cav1.2 to autophagosomes. These findings suggest that autophagy induces atrial electrical remodeling via ubiquitin-dependent selective degradation of Cav1.2 and provide a novel and promising strategy for preventing AF development.

## Introduction

Autophagy, a finely regulated bulk degradation pathway, sequesters a portion of damaged proteins and organelles to maintain cellular homeostasis, particularly in long-lived cells^1^. This process involves engulfing of cytoplasmic constituents within the double-membrane vacuoles designated autophagosomes which subsequently fuse with lysosomes for further degradation^2^. Growing evidence has proved that autophagic flux is critical for the maintenance, adaptation, and repair of myocardium at basal level under normal conditions, whereas excessive autophagy can induce cardiac remodeling in response to stress^3–5^. Autophagic machinery contains autophagy-related (ATG) protein constituents, among which ATG7 acts as an E1-like activating enzyme facilitating the generation of microtubule-associated protein light chain 3 (LC3)-phosphatidylethanolamine (PE)^6^. Numerous works have uncovered that ATG7-mediated autophagy participates in neoplastic, neuronal, and cardiovascular diseases^7–9^. Our previous study has discovered the core autophagy marker LC3B is highly expressed in atrial tissues of chronic atrial fibrillation (AF) patients and canine models with rapid atrial pacing (RAP)^10^. However, the precise role of autophagy in atrial electrophysiology and the mechanism remain poorly understood.

AF is a common sustained atrial arrhythmia in clinical settings and associated with pronounced mortality and morbidity^11^. AF performs self-perpetuating as a consequence of atrial rapid rate, eventually causing electrical remodeling and structural damage. The suboptimal effectiveness of current therapeutic options has fueled further research on the molecular mechanisms underlying the pathogenesis of this arrhythmia^12,13^. Atrial electrical remodeling is a central substrate responsible for AF, which is characterized by abbreviation of action potential duration (APD)^14^. Decreased expression of L-type calcium channel (Cav1.2) along with reduced Ca^2+^ current (*I*_Ca,L_) is the major pathological change in rate-dependent shortening of APD^15^. Recent studies report that ischemia-induced autophagy leads to the selective degradation of Connexin43 with the concomitant recruitment of ubiquitin–proteasome system (UPS) at the intercalated discs, suggesting that autophagy is involved in the biological regulation of transmembrane protein^16,17^. In addition, the crosstalk between ubiquitination and autophagy plays a vital role in the degradation of cellular substrates in response to cardiac stress^18,19^.

The present study was undertaken in an attempt to assess whether autophagic flux is entirely activated in synthesis or impaired in degradative stage during clinical and experimental AF, whether autophagy is implicated in atrial electrical remodeling, and whether a synergic role for autophagy and UPS triggers the selective degradation of Cav1.2 in atrial myocytes to promote AF.

## Results

### Atrial autophagic flux is activated in response to AF

**Fig. 1:**
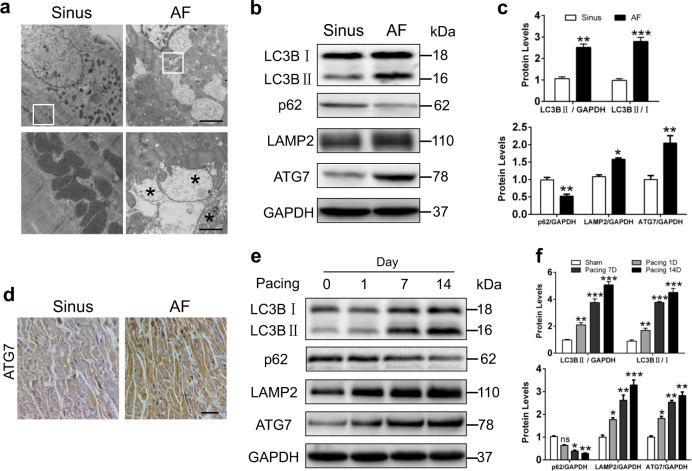
Autophagy-related protein expression in human and pacing rabbit atrial tissues. **a** Representative EM images (original magnification, 8000× and 20,000×) showed double membranes and autophagic vesicles (asterisk) in atrial cardiomyocytes of AF patients. Scale bar: 500 nm, *n* = 5 samples/group. **b** and **c** Western blot showed increased LC3B, p62, LAMP2, and ATG7 expressions in left atrial tissues of AF patients. ^*^P <0.05; ^**^P <0.01; ^***^P <0.001 AF vs. Sinus. **d** Representative immunohistochemical images of ATG7 protein in human atria (original magnification, 100×). Scale bar: 50 μm, *n* = 5 samples/group. **e** and **f** Representative immunoblots for LC3B, p62, LAMP2, ATG7, and GAPDH in left atrial tissues of pacing rabbits. ^**^P <0.01; ^***^P <0.001 vs. Sham

To identify the level of autophagic flux in humans, left atrial appendages were obtained from patients with sinus rhythm (SR) or persistent AF who underwent mitral valve replacement. EM analysis exhibited more autophagosomes formed in AF patients; yet, no obvious autophagic vacuoles were observed in sinus group (Fig. 1a, Supplemental Figure 1a). Then we detected the levels of autophagy-related protein and found a significant increase in LC3BII and LAMP2 expressions with decreased p62 expression (Fig. 1b, c, Supplemental Figure 2), which suggested atrial autophagic activation in AF patients. Besides, we measured the expression of genes closely related to autophagy including *ATG1*, *3–8*, *12*, and *13*. The *ATG7* mRNA levels were significantly upregulated in parallel with higher ATG7 protein in AF group (Supplemental Figure 1b), indicating ATG7 as an essential regulator for activating ATG8 (encoding for LC3) under AF condition. The immunohistochemistry revealed more ATG7 expression in atrial myocardial fibers with AF (Fig. 1d). Moreover, the expressions of ATG5 and BECN1 (ATG6) were unaltered in both groups (Supplemental Figure 1c). The evidence indicated that atrial autophagic flux was activated in the atria of persistent AF patients. Consistently, Western blot analysis showed a gradual increase in the expressions of LC3BII, LAMP2, and ATG7, accompanied with the reduction of p62 in RAP rabbit model (Fig. 1e, f, Supplemental Figure 3). Furthermore, ATG7 mRNA level showed a progressive increase from 1 day to 14 days (Supplemental Figure 4). Taken together, these data demonstrated that atrial autophagic flux was activated in response to AF

.

### ATG7-dependent autophagy contributes to atrial electrical remodeling

**Fig. 2:**
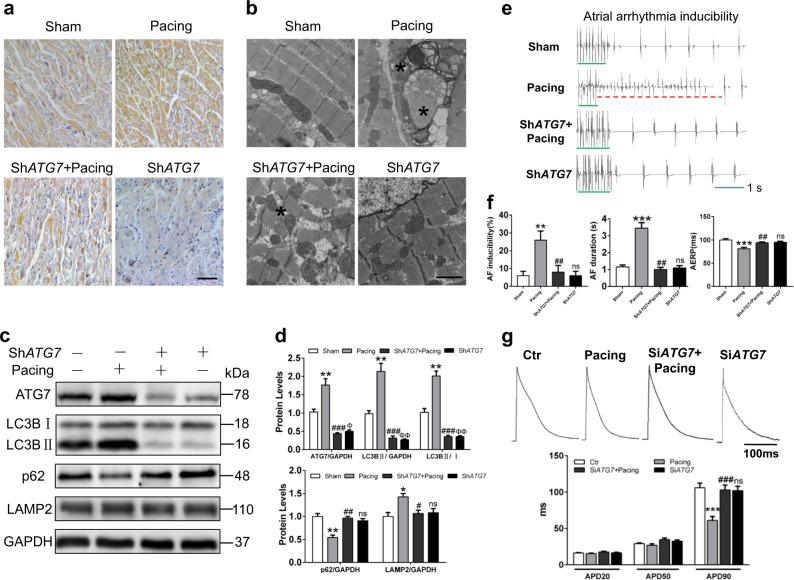
Effects of inhibited autophagy by *ATG7* knockdown on AF vulnerability. **a** Representative immunohistochemical images of ATG7 protein in atrial tissues of rabbits (original magnification, 100×). Scale bar: 50 μm, *n* = 5 samples/group. **b** Electron microscopy showing autophagosomes and degradative autophagic vacuoles (asterisk) in rapid atrial pacing rabbits, and autophagosomes in Pacing + Sh*ATG7*. Scale bar: 500 nm, *n* = 4 samples/group. **c** and **d** Immunoblot of ATG7, LC3B, p62, and LAMP2 in rabbit atrial tissues. ^*^P <0.05, ^**^P <0.01; ^***^P <0.001 vs. Sham. ^#^*P <*0.05; ^##^*P <*0.01; ^###^*P <*0.001 Pacing vs. Sh*ATG7* + Pacing. ^φ^*P <*0.01; ^φφ^*P <*0.05; ns Sham vs. Sh*ATG7*. **e** and **f** The atrial ECG of rabbits. Green line: indicated burst pacing; red line: indicated the incidence of AF. Knockdown *ATG7* reduced inducibility of AF, prolonged duration of AF, and shortened AERP. ^**^P <0.01, ^***^P <0.001 Sham vs. Pacing; ^##^*P <*0.01 Pacing vs. Sh*ATG7* + Pacing; ns Sh*ATG7* vs. Sham. **g** APD recording of HL-1 atrial cardiomyocytes. ^***^P <0.001 vs. control, ^###^*P <*0.001 Pacing vs. si*ATG7* + Pacing, ns control vs. si*ATG7* (*n* = 6 cells/group)

To exactly elucidate the role of autophagy in atrial electrical remodeling, we generated lentiviruses carrying *ATG7* shRNA and injected the lentiviruses into the atrium at multiple locations. Quantitative reverse-transcription polymerase chain reaction (qRT-PCR) analysis and immunohistochemistry staining confirmed significantly decreased ATG7 expression in *ATG7* knockdown group (Supplemental Figure 5a and b, Fig. 2a). To assess whether cardiac function was affected by *ATG7* knockdown, echocardiography and H&E staining were performed. No cardiac dysfunction and structural damage were observed in RAP rabbits injected with *ATG7* knockdown lentiviruses compared with tachypacing group (Online Table 2, Supplemental Figure 5c). Then we found that more degradative autophagic vacuoles were present in RAP rabbits compared with those injected with the *ATG7* shRNA in electron microscope (EM) images (Fig. 2b, Supplemental Figure 5d). Western blot results showed that Sh*ATG7* effectively inhibited the activation of atrial autophagy during AF (Fig. 2c, d). To evaluate whether inhibition of autophagic activity could affect atrial electrical remodeling, an intracardiac electrode was used to detect atrial electrophysiology. *ATG7* knockdown significantly restored atrial effective refractory period (AERP) shortening and reduced the incidence and duration of AF induced by RAP (Fig. 2e, f). Similar to the in vivo observation, tachypacing induced a remarkable abbreviation in the APD of HL-1 cells, which could be reversed by the suppression of autophagic flux via *ATG7* siRNA (Fig. 2g, Supplemental Figure 6a–d). Considering the important role of atrial fibrosis in the pathophysiology of AF, we examined whether modulating ATG7 affected atrial fibrosis. Atrial fibrosis levels did not differ in the presence or absence of *ATG7* knockdown (Supplemental Figure 5e and f). These data demonstrated that inhibition of autophagy could attenuate atrial electrical remodeling and reduce AF susceptibility induced by RAP.

**Fig. 3:**
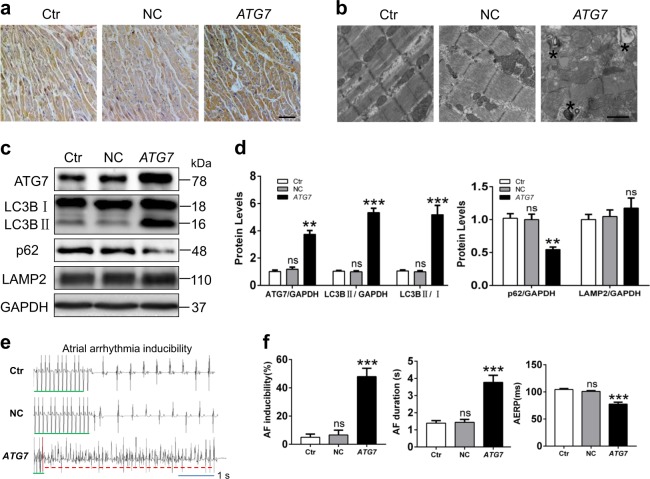
Effects of increased autophagy via *ATG7* overexpression on AF vulnerability. **a** Representative immunohistochemical images of ATG7 protein in atrial tissues of rabbits (original magnification, 100×). Scale bar: 50 μm, *n* = 5 samples/group. **b** Electron graphs of atrial tissues showing autophagosomes and degradative autophagic vacuoles (asterisk) in *ATG7* group. Scale bar: 500 nm, *n* = 5 samples/group. **c** and **d** Immunoblots of ATG7, LC3B, p62, and LAMP2 expressions in rabbit atrial tissues; ns Ctr vs. NC, ^***^P <0.001 Ctr vs. *ATG7*. **e** and **f** The representative ECG of rabbits by atrial burst pacing. The statistical data of AF inducibility, AF duration, and AERP in three groups; ns Ctr vs. NC, ^***^P <0.001 Ctr vs. *ATG7*

We next investigated whether *ATG7* overexpression promoted AF development. qRT-PCR revealed upregulation of *ATG7* mRNA in *ATG7* overexpressed rabbits compared with control and negative lentiviral (NC) animals (Supplemental Figures 7a and  3a). The initial autophagic vacuoles and autophagic cargos with high electron density reflected that both autophagosome generation and lysosomal degradation were activated in *ATG7* group (Fig. 3b and Supplemental Fig. 7b). Furthermore, Western blot showed increased ATG7 and LC3BII expression implying activated autophagy, similar to immunohistochemistry results (Fig. 3c, d). As expected, our data confirmed that *ATG7* overexpression promoted AF vulnerability and shortened AERP (Fig. 3e, f). However, *ATG7* overexpression did not worsen atrial physiological function and structure assessed by echocardiography and H&E staining (Online Table 3; Supplemental Figure 7c). Masson staining and collagen I and collagen III expressions had no difference among the three groups (Supplemental Figure 7d–f). These findings implied that *ATG7* overexpression-induced autophagy could shorten AERP and contribute to the occurrence and maintenance of AF.

### Activated autophagy diminishes Ca^2+^ current and degrades Cav1.2 protein

**Fig. 4:**
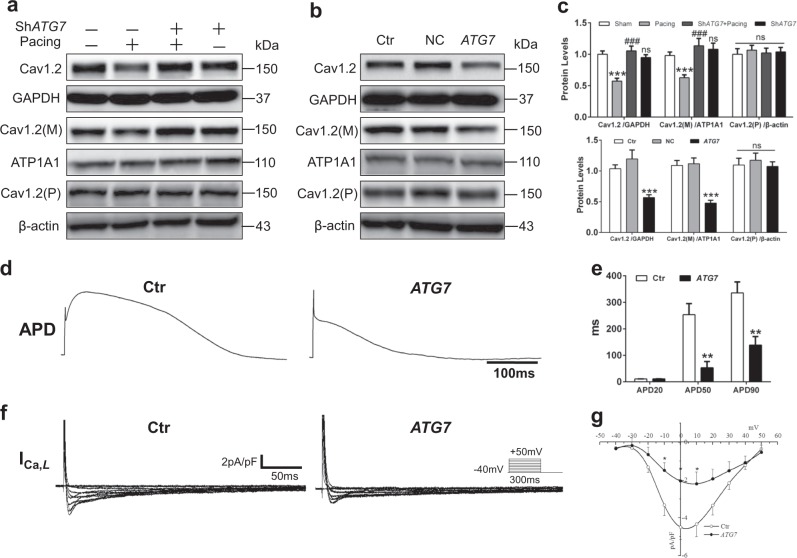
Cav1.2 protein and *I*_Ca,L_ were decreased by activated autophagy. **a** and **c** Immunoblot of total, cellular membrane and plasma Cav1.2 protein in rabbit atrial tissues. ^***^P <0.001 Sham vs. Pacing; ^###^*P <*0.001 Pacing vs. Sh*ATG7* + Pacing; ns Sham vs. Sh*ATG7*. **b** and **c** Immunoblot of total, cellular membrane and plasma Cav1.2 protein in *ATG7* rabbit atrial tissues. ^***^P <0.001 Ctr vs. *ATG7*. **d** and **e** The APD records of control and overexpression *ATG7* cardiomyocytes isolated from rabbit atria, ^**^P <0.01 Ctr vs. *ATG7*, *n* = 6 cells/group. **f** and **g** Ca^2+^ current reduced in *ATG7* cardiomyocytes, *n* = 6 cells/group

Previous studies have reported that decreased calcium ion channel, especially the L-type channel (Cav1.2), is a well-known feature for AF development. Therefore, we respectively detected total, cytoplasmic, and cellular membrane Cav1.2 expression in atrial tissues of AF patients, and used GAPDH, ATP1A1, and β-actin as protein expression control. The results showed the reduced expression of total and plasma membrane Cav1.2 protein accompanied by unchanged cytoplasmic Cav1.2 protein (Supplemental Figure 8a and b). In addition, total and membrane Cav1.2 proteins were significantly downregulated in pacing rabbits and reversed by *ATG7* knockdown (Fig. 4a and Supplemental Figure 9a). In contrast, the membrane Cav1.2 protein was reduced by *ATG7* overexpression, while the cytoplasmic Cav1.2 protein was unchanged in these three groups (Fig. 4b, c; Supplemental Figure 9b). We next measured the APD and *I*_Ca,L_ in atrial cardiomyocytes isolated from control and *ATG7* overexpression rabbits. The APD_50_ and APD_90_ in *ATG7* overexpression rabbits were significantly shortened and *I*_Ca,L_ density was substantially decreased compared with control ones (Fig. 4d–g). However, *ATG7* overexpression-induced autophagy did not alter the expression of Kir2.1, Kv1.5, Kir3.4, and Kv4.3 (ion channels related AF) as shown by immunoblots (Supplemental Figure 10a and b). These data indicated that Cav1.2 may be a critical target for autophagy regulation of atrial ion channel.

**Fig. 5:**
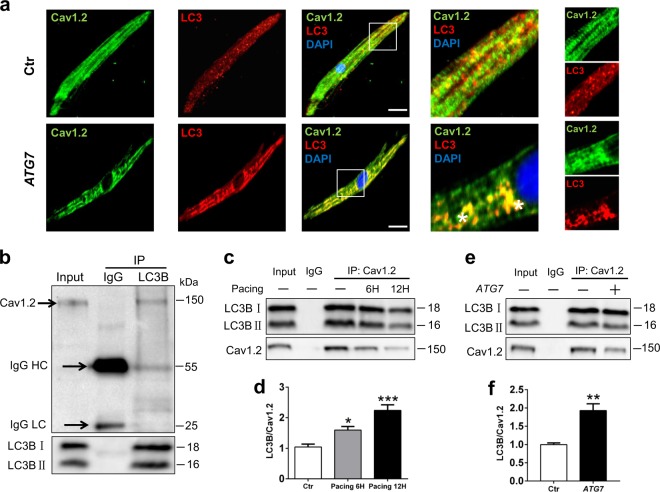
Co-localization and interactions of Cav1.2 with LC3B-positive autophagosomes. **a** Immunofluorescence images showing more co-localization between Cav1.2 and LC3B in *ATG7* group. Scale bar: 25 µm. **b** Fractions of HL-1 atrial cardiomyocytes were immunoprecipitated with anti-LC3 antibody, and immunoblotting was performed with anti-Cav1.2 antibody. **c** and **d** The interaction between Cav1.2 and LC3B in HL-1 cells under *ATG7* overexpression was increased via co-immunoprecipitation. ^*^P <0.05 Ctr vs. Pacing 6 h, ^***^P <0.001 Ctr vs. Pacing 12 h. **e** and **f** Tachypacing-induced increased interaction between Cav1.2 and LC3B in HL-1 cells via co-immunoprecipitation analysis. ^**^P <0.01 Ctr vs. *ATG7*

Then we performed immunofluorescence analysis and observed greater combination between Cav1.2 and LC3B in the atrial tissues of *ATG7* overexpression rabbits (Supplemental Figure 11a and b). Also, the co-localization was increased in atrial cardiomyocytes isolated from *ATG7* overexpression rabbits compared with control ones (Fig. 5a and Supplemental Figure 11c). The co-localization between the two proteins was increased in the pacing group, while *ATG7* knockdown could effectively inhibit the above phenomenon (Supplemental Figure 12a and b). To confirm whether autophagy-induced degradation of Cav1.2 involved the recruitment of Cav1.2 to LC3-positive autophagosomes, we immunoprecipitated Cav1.2 and tested its interaction with LC3. As expected, our data identified that LC3B co-immunoprecipitated with Cav1.2 in normal condition, indicating that autophagosomes interacted with *I*_Ca,L_ α-subunits (Fig. 5b). In addition, the amount of LC3B co-immunoprecipitated with Cav1.2 protein was further enhanced by *ATG7* overexpression (Fig. 5c, d). Similar to the above results, the interaction of Cav1.2 with LC3B increased in parallel with the time of pacing (Fig. 5e, f). In summary, these data indicate that activated autophagy induces the recruitment of Cav1.2 to LC3B-positive autophagosomes, which promotes selective degradation of Cav1.2.

### Ubiquitination as a synergetic signal for Cav1.2 degradation with autophagy

**Fig. 6:**
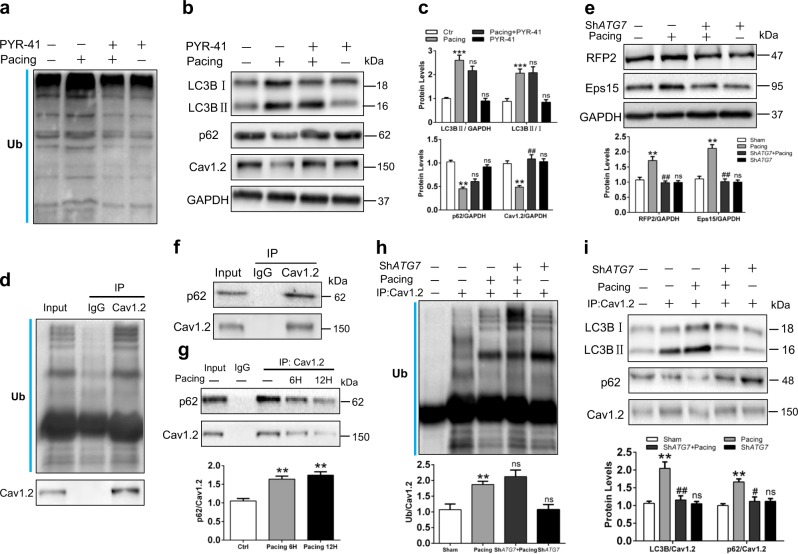
Ubiquitination as a signal for Cav1.2 degradation by autophagy. **a**–**c** Ubiquitin and autophagy-related protein expressions under pacing with or without PTR-41 (E1 inhibitor) in HL-1 cells. ^**^P <0.01; ^***^P <0.001 Ctr vs. Pacing; ^##^*P <*0.01 Pacing vs. Pacing + PYR-41; ns Ctr vs. Pacing + PYR-41. **d** Fractions of HL-1 cells were immunoprecipitated with Cav1.2 antibody, and Western Blot was performed with Ub antibody. **e** Immunoblots of RFP2 and Eps15 under *ATG7* knockdown. ^**^P <0.01 Sham vs. Pacing, ^##^*P <*0.01 Pacing vs. Pacing + Sh*ATG7*, ns Sham vs. Sh*ATG7*. **f** and **g** Fractions of HL-1 atrial cardiomyocytes were immunoprecipitated with anti-p62 antibody, and immunoblot was performed with anti-Cav1.2 antibody. Tachypacing-induced increased interaction between Cav1.2 and p62 in HL-1 cells via co-immunoprecipitation analysis. **h** and **i** Cav1.2 interacted with Ub, LC3B, and p62 following Sh*ATG7* via co-immunoprecipitation. ^**^P <0.01 Sham vs. Pacing; ^#^*P <*0.05, ^##^*P <*0.01 Pacing vs. Pacing + Sh*ATG7*; ns Sham vs. Sh*ATG7*

Several studies have reported that ubiquitin could be bound to cellular components to mediate their delivery into phagophores for facilitating autophagy-dependent degradation^16^. Therefore, we tested the level of ubiquitin in atrial tissues of AF patients and found increased ubiquitin under AF conditions, similar to previous studies (Supplemental Figure 13a). To clarify the specific mechanism of the interaction between LC3B-autophagosome and Cav1.2, PYR-41 (the E1 Ub-activating enzyme inhibitor) was applied to confirm whether ubiquitin is attached to Cav1.2 for facilitating autophagic degradation. Our data showed that Cav1.2 interacted with ubiquitin and the decreased expression of Cav1.2 was blocked by PYR-41 in tachypaced HL-1 cells (Fig. 6a–d), suggesting ubiquitin as a key signal for tachypacing-induced autophagic clearance of Cav1.2. Previous studies identify that RFP2, an E3 ligase, is required for mediating the conjugation of ubiquitination to Cav1.2 and can associate with Cav1.2^20^. Therefore, we detected whether RFP2 is involved in Cav1.2 ubiquitination under AF conditions. The results showed that tachypacing promoted the elevation of RFP2 protein, which could be reversed by *ATG7* knockdown. Then we investigated the role of Ub-binding protein Eps15, participating in endocytosis process of ubiquitinated aggregates. The data showed that Eps15 expression was increased in AF and could be inhibited by *ATG7* knockdown (Fig. 6e). Moreover, the levels of RFP2 and Eps15 were increased in the atrial tissues of *ATG7* overexpression rabbits (Supplemental Figure 13b and c). It is widely accepted that autophagy receptors, such as p62 and NBR1, recognize the ubiquitinated substrates for entry into autophagosomes to mediate the degradation of the targets. Therefore, we evaluated whether p62 could associate with Cav1.2 channel in AF. The data showed that p62 co-immunoprecipitated with endogenous Cav1.2 channels and the interaction was significantly enhanced under tachypacing conditions in HL-1 atrial cardiomyocytes (Fig. 6f, g). Furthermore, the interaction of Cav1.2 with p62 and LC3B significantly enhanced in pacing rabbits atrial tissues, and accompanied with marked ubiquitinated Cav1.2. Conversely, the amount of p62 and LC3B that co-immunoprecipitated with Cav1.2 diminished when autophagy was prevented by *ATG7* knockdown (Fig. 6h, i). These results suggest that autophagic degradation of Cav1.2 requires ubiquitin to trigger the recruitment of LC3B-positive autophagosomes via RFP2 and p62.

### Chloroquine attenuates atrial autophagic flux to prevent AF

**Fig. 7:**
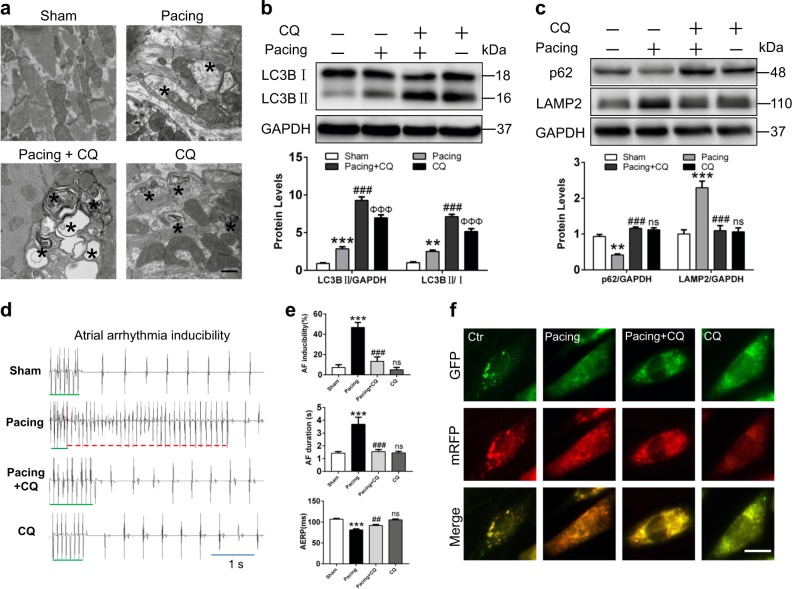
CQ attenuated the vulnerability of AF via inhibiting autophagy. **a** Representative EM images (original magnification, 20,000×) in atrial tissues. Arrow points autophagosomes and autophagic vacuoles. Scale bar: 500 nm, *n* = 5 samples/group. **b** and **c** Representative immunoblots for LC3B, p62, LAMP2, and GAPDH with and without CQ. ^*^P <0.05; ^**^P <0.01; ^***^P <0.001 vs. Sham. ^###^*P <*0.001 Pacing vs. Pacing + CQ. ^φφφ^*P <*0.001; ns Sham vs. CQ. **d** and **e** ECG of Sham, Pacing, Pacing + CQ, and CQ-treated rabbits. The statistical data showing the inducibility, duration, and AERP induced by burst pacing in rabbits. ^***^P <0.001 Sham vs. Pacing, ^##^*P <*0.01, ^###^*P <*0.001 Pacing vs. Pacing + CQ; ns Sham vs. CQ. **f** Representative immunofluorescence images of GFP-mRFP-LC3 dots in HL-1 atrial cardiomyocytes. Scale bar: 25 µm. *n* = 10 cells/group, five independent experiments. ^***^P <0.001 vs. Sham, ^###^*P <*0.001 Pacing vs. Pacing + CQ. ^φ^*P <*0.05 Sham vs. Ctr

Chloroquine (CQ), a lysosome inhibitor, can block the fusion of autophagosomes with lysosomes to inhibit autophagic flux and serve as an anti-inflammation drug in the clinic. Therefore, we explored whether CQ could prevent AF via modulating atrial autophagy. The electron graphs exhibited that degradative autophagic vacuoles (containing partially digested contents) were predominant in pacing atrium; however, more accumulated autophagic structures were present under pacing condition plus CQ (Fig. 7a and Supplemental Figure 14a). In accordance with the above results, CQ pretreatment resulted in further upregulation of LC3BII and p62 proteins compared with RAP rabbits (Fig. 7b, c). Similar results were obtained from tachypaced HL-1 cells by mRFP-GFP-tagged LC3 system. Tachypacing caused increased yellow and red dots in merged images, indicating the activation of both autophagosome formation and lysosome degradation in AF. Whereas CQ pretreatment resulted in accumulation of yellow dots, which implied impaired auto-lysosomal degradation (Fig. 7f and Supplemental Figure 14b). H&E and Masson staining revealed no obvious structural derangement in pacing atrium compared with CQ group (Supplemental Figure 14c and d), indicating that CQ had no structural damage on atrial tissues of rabbits. Then we found that CQ could effectively overcome autophagy-induced AF vulnerability (Fig. 7d, e). Furthermore, CQ treatment restored the degradation of Cav1.2 resulting from tachypacing-induced autophagic flux (Supplemental Figure 15a and b). These results demonstrated that CQ inhibited the promotion of AF via blocking autophagy process and may serve as a therapeutic target in clinic.

## Discussion

**Fig. 8:**
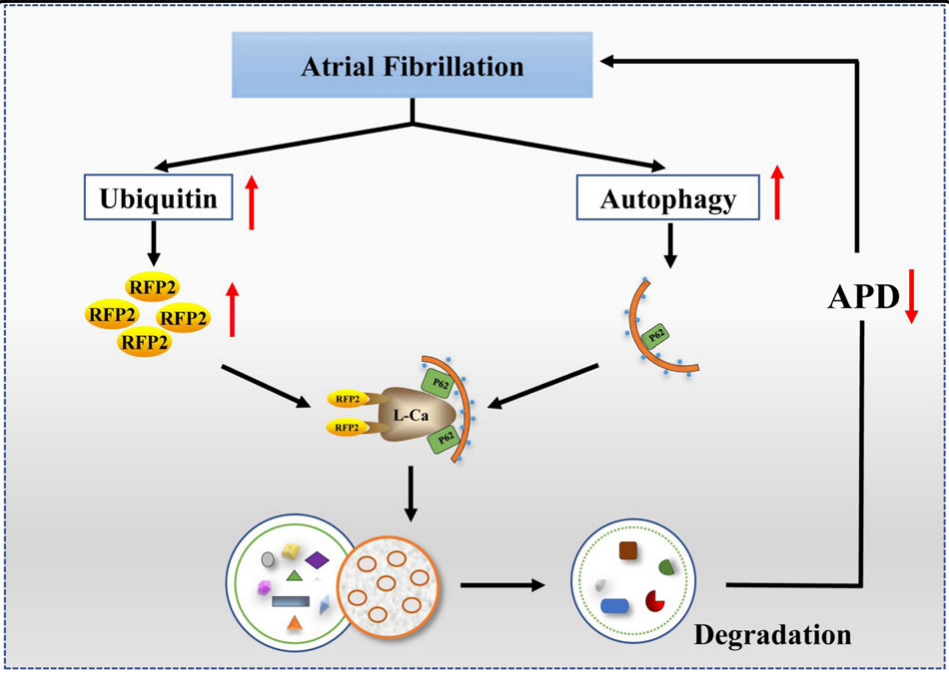
Schematic figure showing autophagy as a novel substrate to promote AF. Autophagy is a bidirectional degradative pathway to clear damaged molecules, lipids, and organelles for maintaining cellular homeostasis. AF triggers ubiquitin and autophagic flux in atrial cardiomyocytes. Ubiquitin as a critical signal mediates delivery of Cav1.2 into autophagosomes via Ub-binding protein RFP2 and p62 in AF. Autophagy contributes to degradation of L-type calcium ion channel, which results in decreased Ca^2+^ currents. Diminished *I*_Ca,L_ reduces APD and increases AF vulnerability

Our data for the first time uncovered a previously unrecognized role of autophagy in AF. It was revealed that activated atrial autophagic flux resulted in AERP shortening and increased AF susceptibility. We then demonstrated that upregulated autophagy by *ATG7* overexpression could induce selective degradation of Cav1.2 protein which caused decreased *I*_Ca,L_. Mechanistically, ubiquitin as a vital signal mediated internalization and interaction of Cav1.2 with LC3B-positive autophagosomes by Ub-binding proteins RFP2 and p62. Taken together, these results suggest that autophagy provides an indispensable substrate for AF inducibility, and indicate autophagic machinery as a potential therapeutic target for AF (Fig. 8).

It is well recognized that autophagy helps to sustain cellular homeostasis and promote survival after oxidative stress or ischemia/hypoxia insult^21^. Although the role of autophagy has been examined in many cardiovascular diseases, such as myocardial ischemia, pathological cardiac hypertrophy, and heart failure^22,23^, the detailed alteration of autophagic flux and its precise effect on AF is unclear. Chen et al. reported that upregulated autophagy occurred in most cases of atrial cardiomyocytes in patients with severe mitral and tricuspid regurgitation with or without AF^24^. In contrast, Garcia et al. claimed that autophagic vesicles accumulated but LC3B expression reduced in atrial tissues from patients developing AF after coronary artery bypass surgery, suggesting impaired autophagic process^25^. Above discrepant results imply that the alternation of autophagic flux may be different in response to diverse environmental stimulates. Our data confirmed that the number of initial and degradative autophagic vacuoles and LC3B and LAMP2 expressions were increased while p62 decreased in atrial samples of persistent AF patients, indicating that autophagic flux was activated. Recently, Marit et al. claimed that activated autophagy of atrial cardiomyocytes correlated with AF progression in tachypaced canines^26^. Consistently, we observed that the autophagic process was actually activated in AF rabbit model by RAP and showed a time-dependent increase. In summary, these data illustrated that the autophagy machinery was stimulated in autophagosome formation and autolysosome combination in both clinical and experimental AF. ATG genes as the necessary promoters can modulate the elongation and expansion of the autophagic phagophores; among these, ATG7 is essential for activating ATG8-PE^6^. Previous studies have uncovered that ATG7 plays a key regulatory role in autophagy in the heart. Guo et al. demonstrated that ATG7 is involved in autophagosome formation induced by acetaldehyde production following alcohol intake and promotes cardiomyocyte contractile dysfunction in murine hearts^27^. In this study, we clearly clarified that ATG7 participated in clinical and experimental AF and induced atrial autophagy. Though morpholino knockdown of *ATG5*, *ATG7*, and *Becn1* leads to aberrant heart development at embryonic stage^28^, our study established *ATG7* knockdown in adult rabbits via atrial injection with lentiviruses and found no remarkable structural damages. These data illustrated the notion that ATG7-mediated autophagy stimulates in AF.

A large body of preclinical evidence suggests that autophagy is a double-edged sword in cardiovascular disease, acting in either beneficial or maladaptive ways, depending on the context^29^. Thus far the role of autophagy in AF is not well established. Our data showed that upregulated autophagic flux by *ATG7* overexpression provoked the incidence and perpetuation of AF; conversely inhibition of autophagy via *ATG7* knockdown and autophagy inhibitor CQ (a lysosomotropic agent that prevents endosomal acidification) could ameliorate AF vulnerability. We also observed that enhanced autophagy contributed to aberrant shortening of AERP suggesting atrial electrical remodeling. In fact, AF is clearly a complex condition that results from multiple potential contributors that can interact with each other^11^. In addition to electrical remodeling and structural alterations, atrial fibrosis also affects the development of AF^30,31^. However, the protective effects against AF promotion that we observed by *ATG7* knockdown and the opposite phenomena with *ATG7* overexpression are not associated with fibrosis process. These results revealed that autophagy played a facilitating role in adverse atrial electrical remodeling and subsequent AF synthesis.

AF causes atrial electrical remodeling, primarily due to rapid atrial rate that promotes AF perpetuation (“AF begets AF”)^14^. APD abbreviation is pivotal in AERP shortening, which is, in turn, a primary factor in AF promotion^32^. It has been widely accepted that *I*_Ca,L_ density reduction resulting from transcriptional downregulation and functional impairment of Cav1.2 is a principal causal factor for the remodeling^33^. Previous studies have indicated that diverse exogenous stimuli, such as inflammation, microRNA, and oxidative stress signals contribute to decreased Cav1.2 expression^14,34^. Our data revealed that upregulation of autophagy recapitulated the major characteristics of Cav1.2 impairment triggered by tachypacing both in vivo and in vitro; in sharp contrast, inhibition of autophagy by *ATG7* knockdown and CQ inhibited the reduction of Cav1.2. In the present study, we discovered that autophagy as a novel substrate exacerbates decreased Cav1.2 along with diminished *I*_Ca,L_ for promoting electrical remodeling.

In addition, we confirmed that the co-localization and interaction between LC3B and Cav1.2 in atrial cardiomyocytes were present in physical condition, which increased during tachypacing and *ATG7* overexpression. Our study also identified that enhanced autophagy significantly induced selective degradation of Cav1.2 with unaltered expression of AF-related potassium ion channels. Existing studies have reported that autophagy can selectively degrade cellular transmembrane protein. Hesketh et al. found that in canine ventricular tissue, internalized gap junctions were incorporated into multilamellar membrane structures with features characteristic of autophagosomes; intracellular connexin43 extensively co-localized with the autophagosome marker GFP-LC3 when both proteins were exogenously expressed in HeLa cells^19^. Ahn et al. reported that transient receptor potential vanilloid type 1 (TRPV1) can be degraded by starvation- and glucocorticoid-mediated autophagy^35^. Similar to the previous studies, we confirmed that Cav1.2 is the marked target combined with LC3-positive autophagosomes for further degradation during AF.

Recently, several studies supported the notion that ubiquitin is a critical signal to mediate molecules delivery into autophagosomes relying on autophagic adaptor proteins^36,37^. Ubiquitin system is complicated in regulating ion channel density in the plasma membrane. Jespersen et al. found that the surface of KCNQ1 is modulated by Nedd4/Nedd4-like-dependent ubiquitylation^38^. Altier et al. proved that RFP2, an E3 ubiquitin ligase, regulates ubiquitination and membrane expression of endogenous Cav1.2 in rat brain homogenate^20^. We discovered that RFP2 expression significantly upregulated and promoted ubiquitination of Cav1.2 during AF. Additionally, the Ub-binding proteins Eps15 and p62 recognized ubiquitinated Cav1.2, then mediated the internalization and transportation of Cav1.2 into autophagosomes. The interaction between Cav1.2 and p62 further induced the selective autophagic degradation of Cav1.2, which was attributed to ubiquitin.

Although we identified autophagy activation as an important contributor to *I*_Ca,L_ downregulation and AF promotion, it does not exclude other mechanisms for governing AF development. First, we only tested the AF-related ion channel proteins under *ATG7* overexpression; however, whether the current of these channels were altered by autophagy need to be further interpreted. Our study could not exclude the effect of potassium current on AF development. The second limitation of our study is that the characteristics of atrial electric remodeling are different among diverse types of underlying heart diseases. Our observations of the AF-promoting effect by activated autophagy obtained from tachypacing induced AF, which should not be extrapolated to other types of AF. Whether autophagy affected electrical remodeling in other causes AF is unclear. Third, the measurements to monitor autophagic elements at different stages of the autophagic process were limited in vivo. LC3-immunogold labeling analysis may be used to clarify the features of autophagic process under AF condition in the subsequent research.

In summary, our work demonstrated that activated autophagic flux is involved in the initiation and perpetuation of AF, and atrial electrophysiology is critically affected by autophagy. These findings highlight the importance of autophagic degradation of L-type Ca^2+^ channel, which provides new insights into biochemical mechanisms of electrical remodeling in AF and indicate autophagy as a potential therapeutic target.

## Materials and methods

### Human atrial tissues

All patients provided written informed consent before enrollment and complied with the principles that govern the use of human tissues outlined in the Declaration of Helsinki. Left atrial appendages were obtained as surgical specimens from patients undergoing cardiac surgery for mitral valve replacement, following established procedures approved by Ethic Committee of the Harbin Medical University. Patients with SR (*n* = 10) and persistent AF (*n* = 12, documented arrhythmia >6 months before surgery). Specimens were immediately stored in liquid nitrogen and transported to the laboratory. The clinical subject characteristics are shown in Online Table 1.

### Animal experiments

All animal handling procedures were approved by the Animal Care and Utilization Committee of Harbin Medical University. The AF animal model was established according to our previous studies^39^. Twenty-one male New Zealand white rabbits were randomly divided into four groups: Sham group (*n* = 6); pacing group (*n* = 5) rapid right atrial pacing (RAP) for 1 week; Sh*ATG7* + pacing (*n* = 5) rabbits were given knockdown *ATG7* lentivirus via multiple-point injection into atrial tissues which were synthesized by Shanghai Genechem (China) along with RAP; Sh*ATG7* group (*n* = 5) knockdown *ATG7* lentivirus injected into atrial tissues. Then, 17 male New Zealand white rabbits were randomly divided into three groups: control operation group (*n* = 6) with PBS injection; NC group (*n* = 6) given negative control lentivirus and enhanced infection solution (Shanghai Genechem China) with multiple-point injection into atrial tissues; *ATG7* group (*n* = 5) *ATG7* lentivirus injected into atrial tissues. Twenty-five male New Zealand white rabbits (2.5–3.0 kg, Experimental Animal Center of the First Affiliated Hospital of Harbin Medical University, Harbin, China) were randomly divided into four groups: Sham group (*n* = 7) with sutured electrodes and no pacing; pacing group (*n* = 6) rapid right atrial pacing for 2 weeks at 600 beats/min; pacing concomitant treatment with CQ 50 mg/kg/day for 2 weeks group (AF + CQ, *n* = 6); CQ group (*n* = 6) given CQ alone (50 mg/kg/day) for 2 weeks. All rabbits need recovery for 3 days after performing operation. A pacemaker (AOO, Fudan University, China) was implanted in a subcutaneous pocket, and connected with an electrode-lead. The atrial electrophysiology was measured as described previously^39^. Successful induction of AF was defined as a period of rapid atrial rhythm lasting at least 1 s. AF vulnerability was determined as the percentage of AF and atrial arrhythmia recorded by an intracardiac electrode sustaining for over 1 s induced by a train of 10 Hz, 2 ms stimuli to the right atrium at four times the threshold current. To measure AERP_200_ at each BCL for all three times, then we obtained the mean value of three AERPs.

### Cell culture and transfection

HL-1 atrial cardiomyocytes were cultured in Claycomb medium (Sigma-Aldrich, USA) with 10% FBS, 1% penicillin/streptomycin, 0.1 mM Norepinephrine (Sigma-Aldrich, USA), and 2 mM L-glutamine (Sigma-Aldrich, USA) at 37 °C in 5% CO_2_. Cells (≥1 × 10^6^ myocytes) were cultured on plates and subjected to tachypacing by the stimulator (YC-2 stimulator), then stimulated at 5 Hz with square pulses of 10 ms duration and a pulse voltage of 1.5 V/cm. The required capture efficiency of 90% cells throughout stimulation. To knockdown *ATG7*, a pool of siRNAs (Invitrogen, USA) was tested for the capacity to silence *ATG7*. The most potent silencing siRNA was selected for subsequent experiments. Cells were transfected with 200 μM siRNA after 24 h plating, and with Lipofectamine 2000 (Invitrogen), in OptiMem (Gibco) media. After transfection, cells were returned to growth media for 6 h. To specifically overexpress *ATG7*, plasmid (Yrbio, China) was constructed. Cells were transfected with 2 μg plasmid after 24 h plating.

### Whole-cell patch-clamp recordings

Patch-clamp techniques were applied to record the APD of HL-1 and rabbit atrial cardiomyocytes. The pipettes of patch electrodes had tip resistances of 2 ~ 3 MΩ when filled with pipette solution. Cells were placed in a 1-ml chamber mounted on an inverted microscope (IX-70; Olympus) and perfused with Tyrode’s solution. *I*_Ca,L_ was measured in atrial cardiomyocytes isolated from rabbit. A holding potential of −80 mV and a 100-ms ramp pulse to −40 mV was used to suppress the Na^+^ current. Whole-cell recording was performed using an Axo-patch 200B amplifier (Axon Instruments). The data were collected and analyzed with the use of pCLAMP 9.2 software.

### GFP-mRFP-LC3

The GFP-mRFP-LC3 lentivirus was purchased from Genechem (Shanghai, China). HL-1 atrial cardiomyocytes cultured on coverslips were transfected with GFP-mRFP-LC3 and control lentivirus and selected with puromycin for a week. The fresh complete medium was changed and cells were viewed under a fluorescence microscope. The number of GFP and mRFP dots was determined by manual counting of fluorescent puncta in five high-power fields (40×, Zeiss, Germany).

### Electron microscopy

Atrial tissues and HL-1 atrial cardiomyocytes were washed using PBS and fixed in 2.5% glutaraldehyde in 0.1 M Cacodylate buffer for 24 h. Post-fixation occurred in 1% osmium tetroxide and 1% aqueous uranyl acetate, respectively, for 1 h. After ascending series of dehydration wit ethanol, the samples were embedded with eponate 12 medium. The sections (75–85 nm) were cut with a Leica Ultramicrotome and a Diatome diamond knife, and post-stained with 5% uranyl acetate for 10 min and lead citrate for 5 min. Philips CM12 electron microscope (FEI Company), operated at 60–120 kV and equipped with a digital camera, was used to image the autophagic vacuoles.

### qRT-PCR

Total RNA was isolated with reagent (Axygen, USA) according to the manufacturer’s instructions. The primers used were listed in Supplementary Information. qRT-PCR (SYBR Green Assay, Roche, Switzerland) was performed on Applied Bio-system. The relative expression levels of mRNAs were calculated and quantified using the 2^−ΔΔCT^ method after normalization with GAPDH.

### Immunofluorescence

Atrial cardiomyocytes isolated from atria were fixed with 4% paraformaldehyde for 30 min and permeabilized with 0.5% Triton X-100 for 15 min. After incubation for 2 h at room temperature with anti-LC3B (CST, 1:100) and anti-Cav1.2 (Abcam, 1:200), cells were washed with 1% PBS. Then they were incubated with secondary antibodies (Beyotime, China, 1:200) for 1 h. Finally, 4′6-diamino-2- phenylindole (DAPI, Beyotime, China) was added to stain the nuclei. Tissues and cells were detected by laser scanning confocal microscope (100×, ZEISS 510S, Germany).

### Co-immunoprecipitation

HL-1 atrial cardiomyocytes and rabbit atrial tissues were resuspended in NP40 lysis buffer and placed on ice for 20 min, and centrifuged at 14,000×*g* for 30 min at 4 °C. The beads were washed with NT-2 (buffer solution) and incubated at 4 °C overnight with primary antibody. Protein G beads were added and incubated for 1 h at 4 °C. The beads were washed five times and boiled for 3 min in 20 μl of 2× SDS loading buffer. Protein complex was separated and detected by immunoblotting analysis.

### Western blot

Western Blot was performed as described previously^39^. Approximately 30–50 μg of proteins were resolved on 8~12% SDS–PAGE and then transferred on polyvinylidene fluoride membranes. Membranes were blocked with 5% non-fat milk for 1 h, and then incubated with primary antibody overnight at 4 °C against the listed antibodies. After washing, the membrane was incubated with the secondary antibody (Santa Cruz Biotechnology, Dallas, USA) for 1 h. Chemiluminescent signals were developed with ECL kit and detected by ChemiDoc XRS gel documentation system (Bio-Rad, Hercules, CA, USA).

### Statistical analysis

Quantative data were expressed as mean ± SEM. An unpaired Student’s *t* test was applied for comparisons in only two groups. One-way analysis of variance (ANOVA) was used for single main-effect-factor experiments. Two-way ANOVA analysis with multiple group comparisons (Bonferroni-corrected Student *t* tests) was used to evaluate two or more main effect factors. The statistical comparisons for AF incidence were performed with *χ*^2^ test. *P* < 0.05 is considered to be significant.

## Electronic supplementary material

Supplemental Figure

Supplemental Data
