# Supplementary material for: Autophagy exacerbates electrical remodeling in atrial fibrillation by ubiquitin-dependent degradation of L-type calcium channel
Source: Cell Death Dis. 2018 Aug 29;9(9):873. doi: 10.1038/s41419-018-0860-y (PMC6115437; doi:10.1038/s41419-018-0860-y)

Supplemental Figure 1

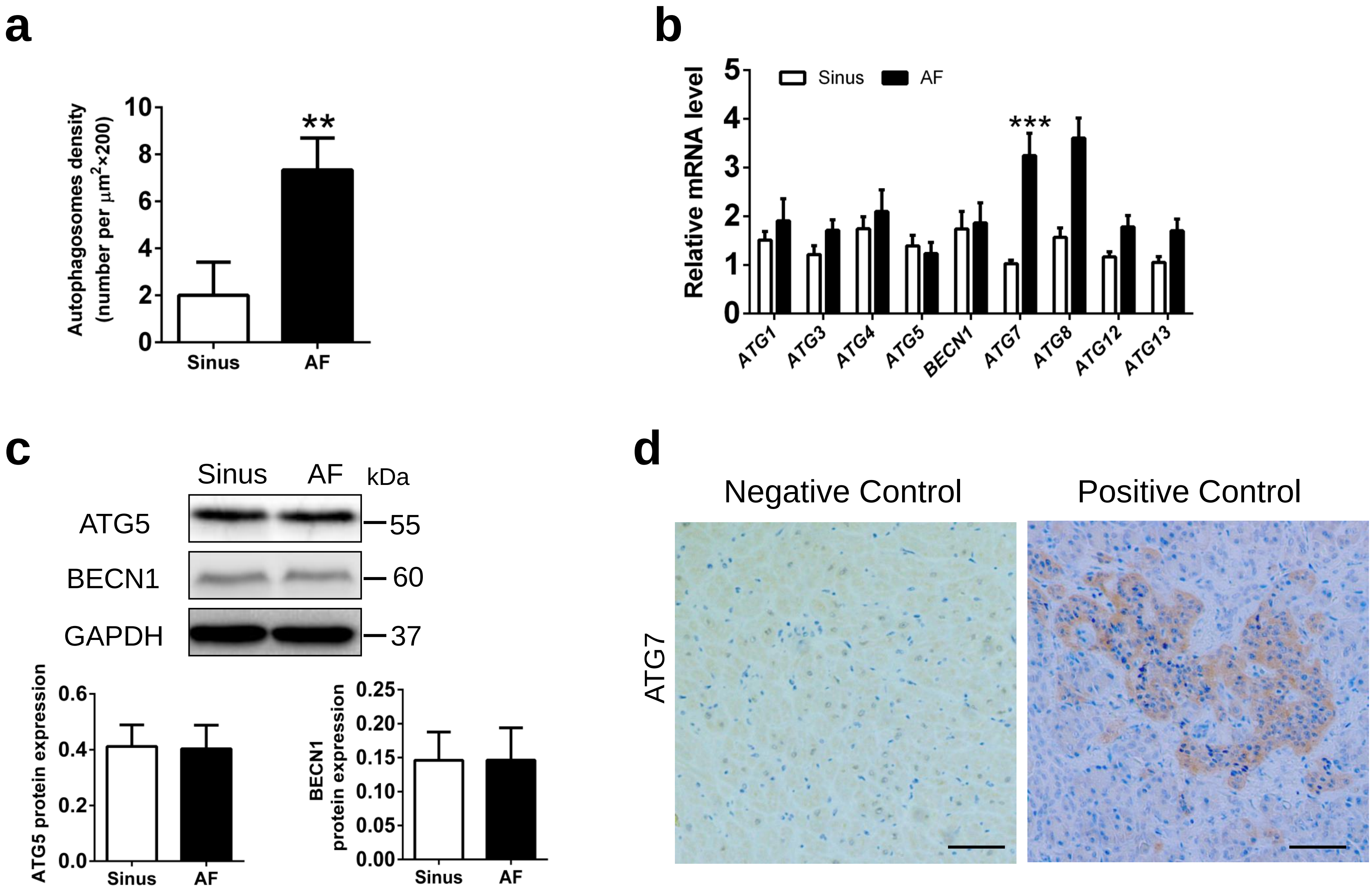

Supplemental Figure 2

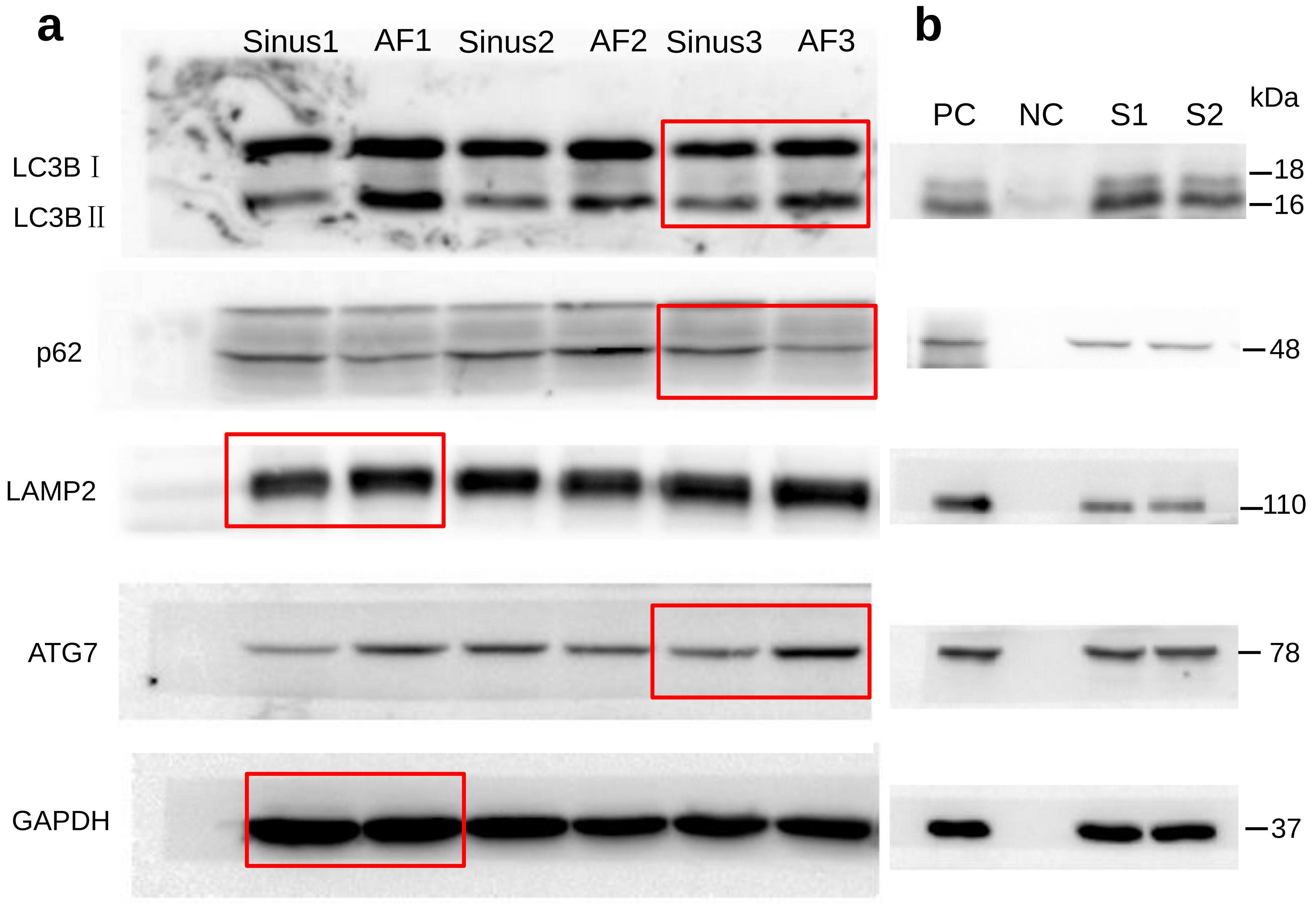

Supplemental Figure 3

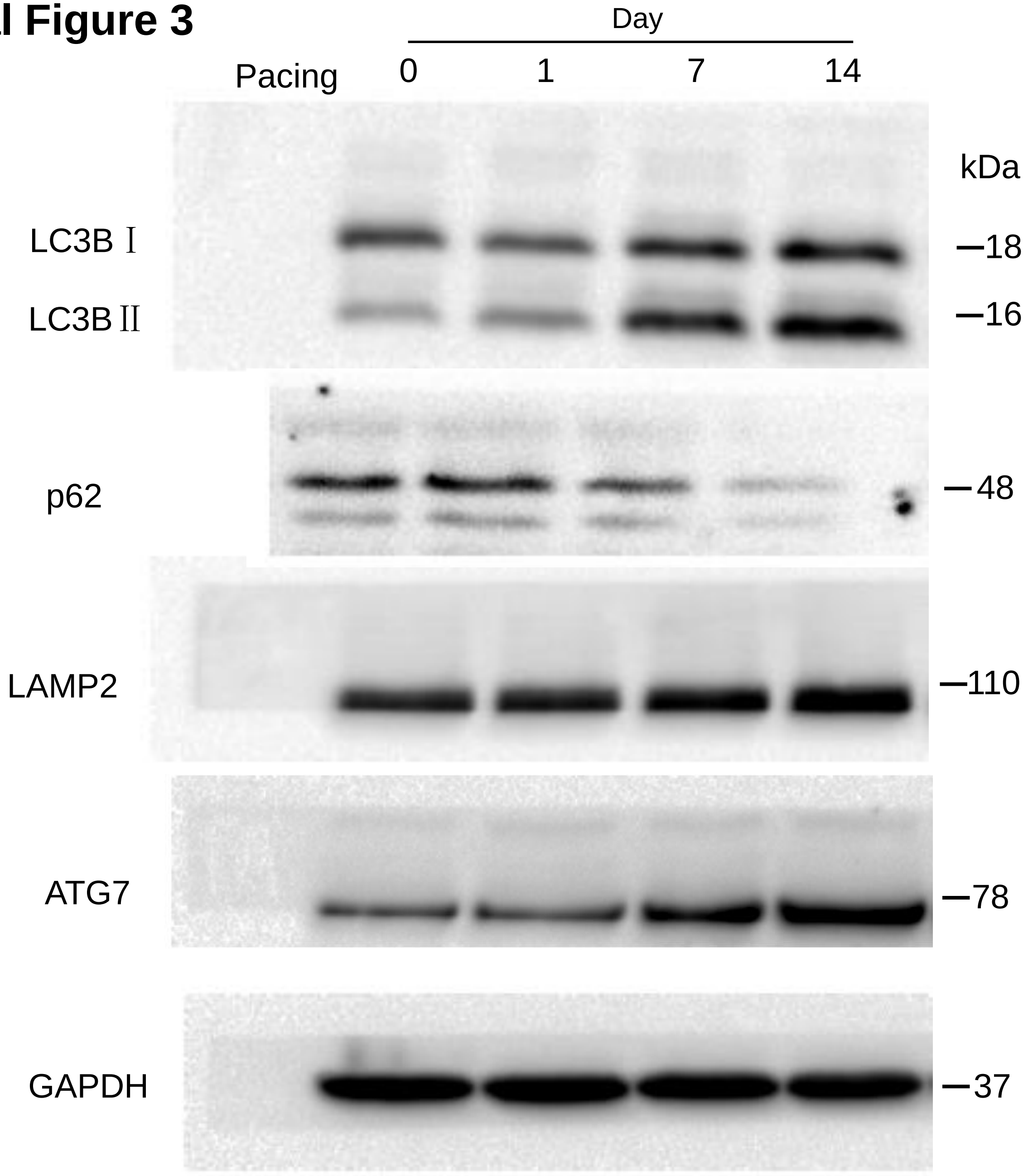

Supplemental Figure 4

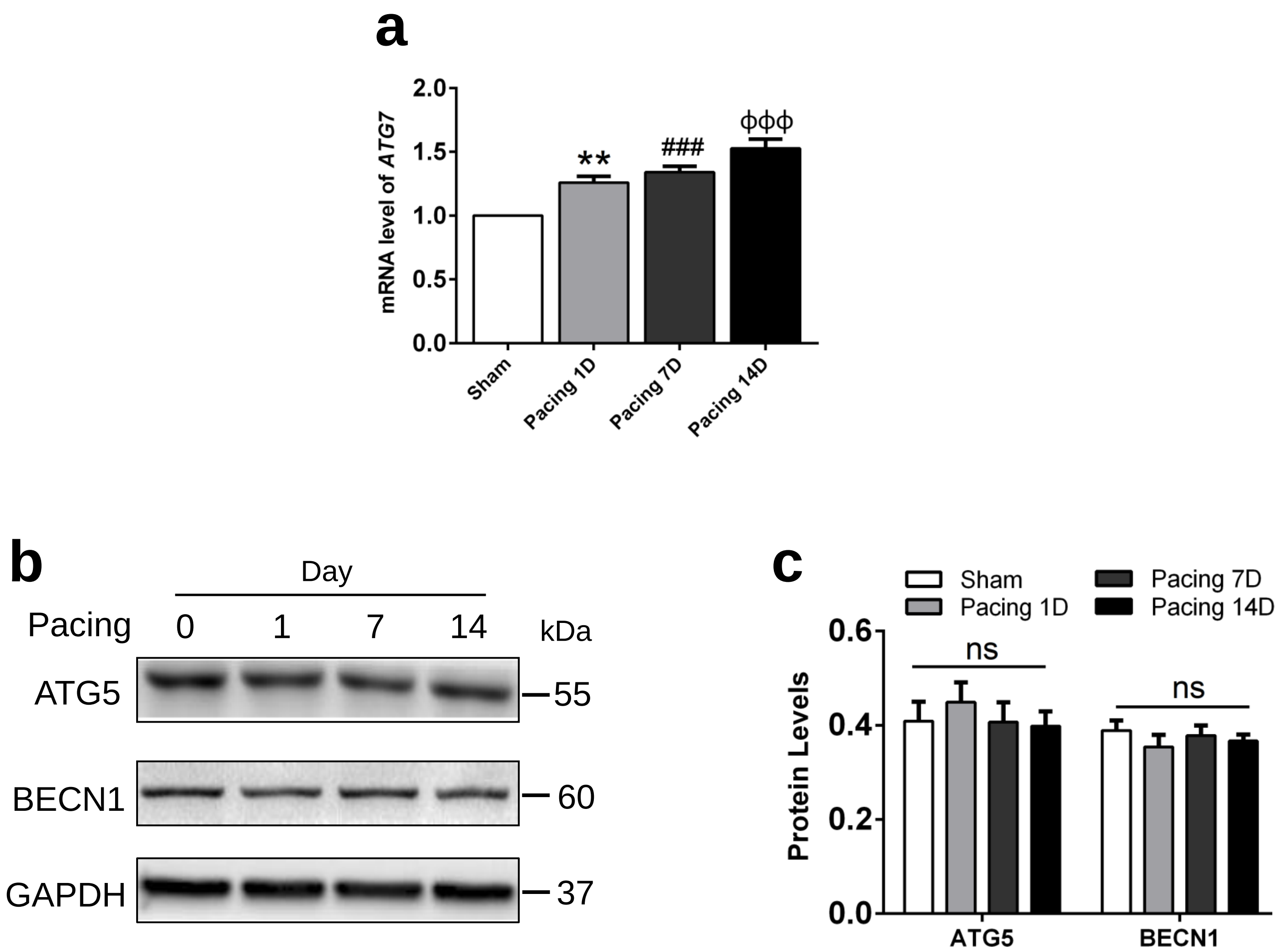

Supplemental Figure 5

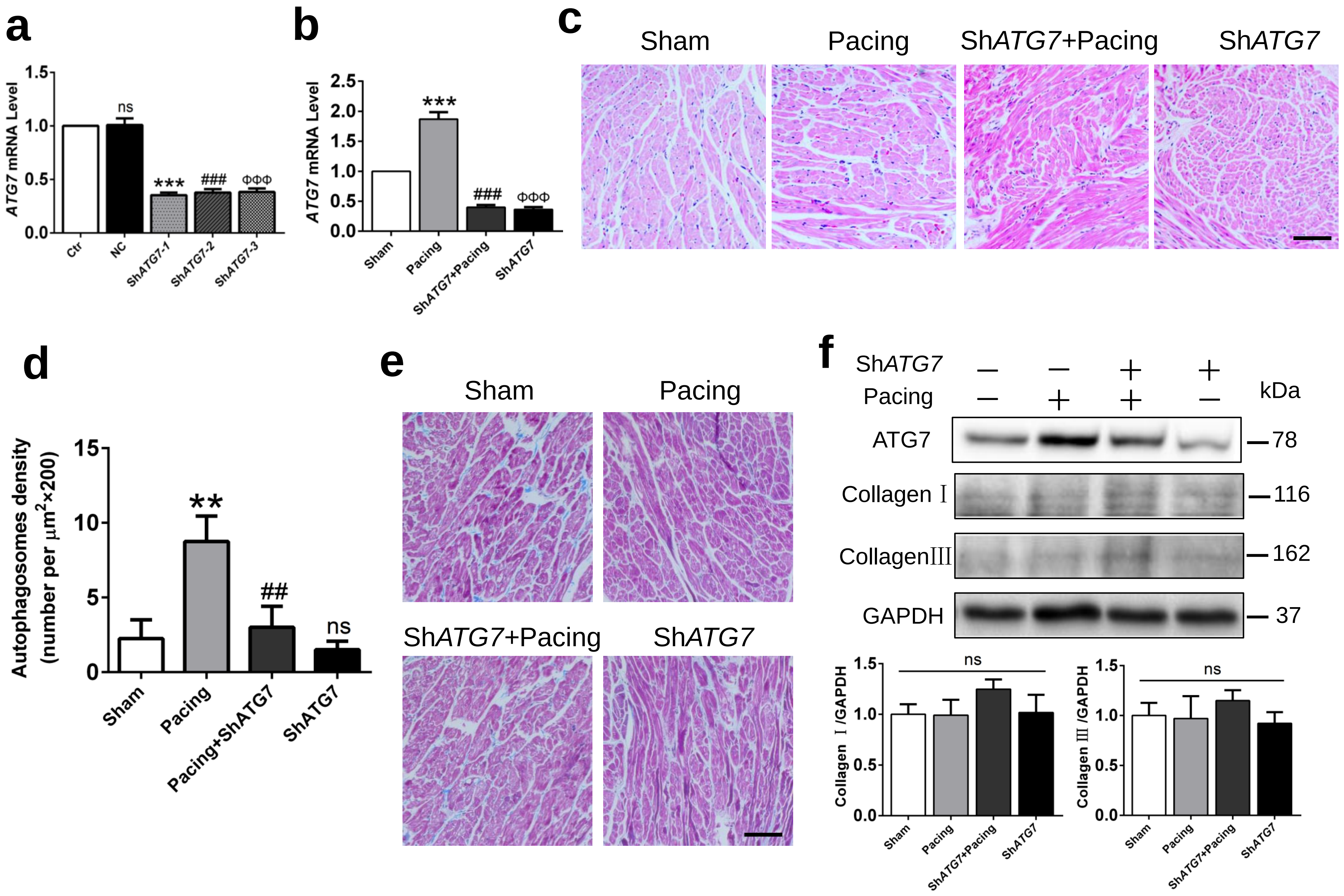

Supplemental Figure 6

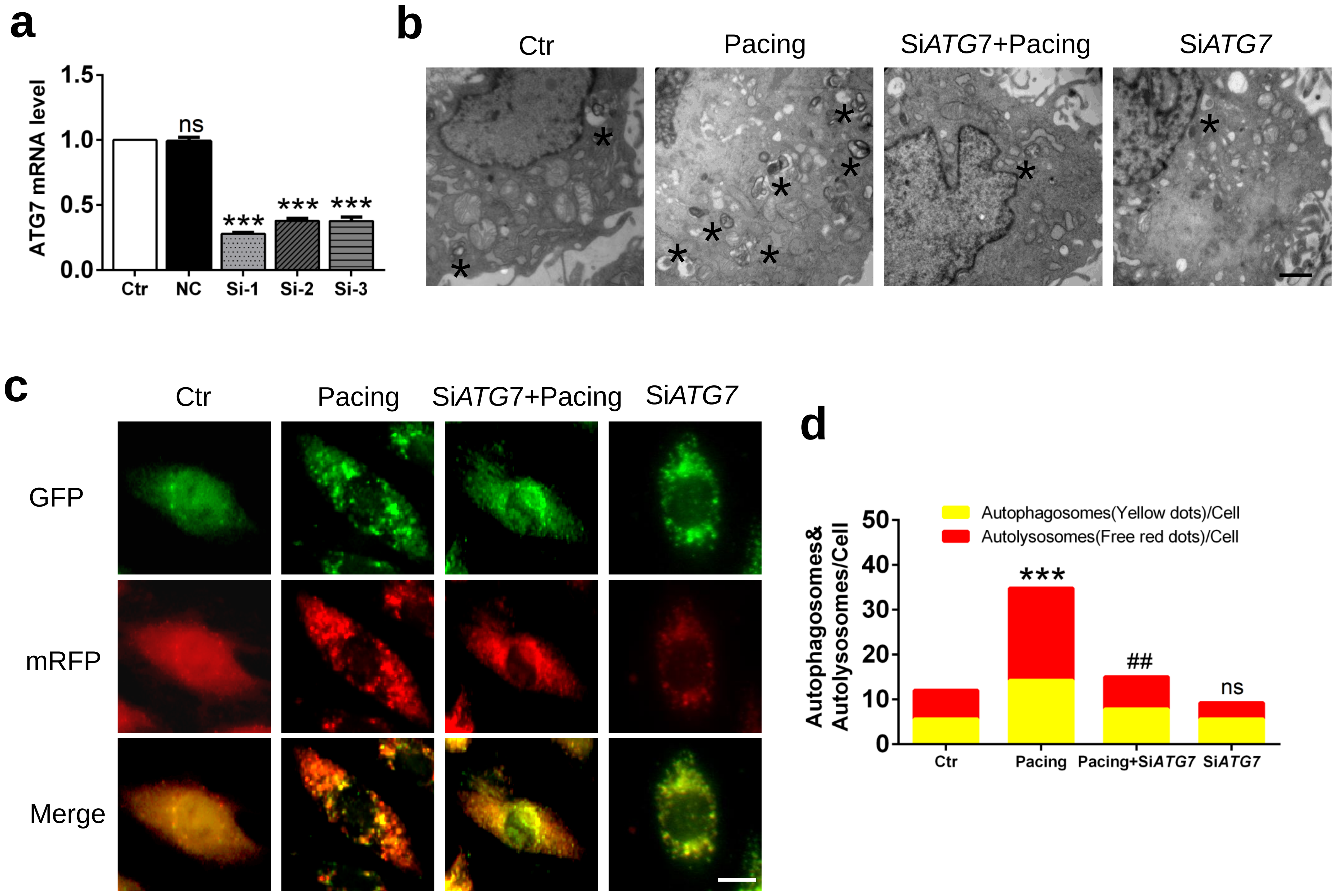

Supplemental Figure 7

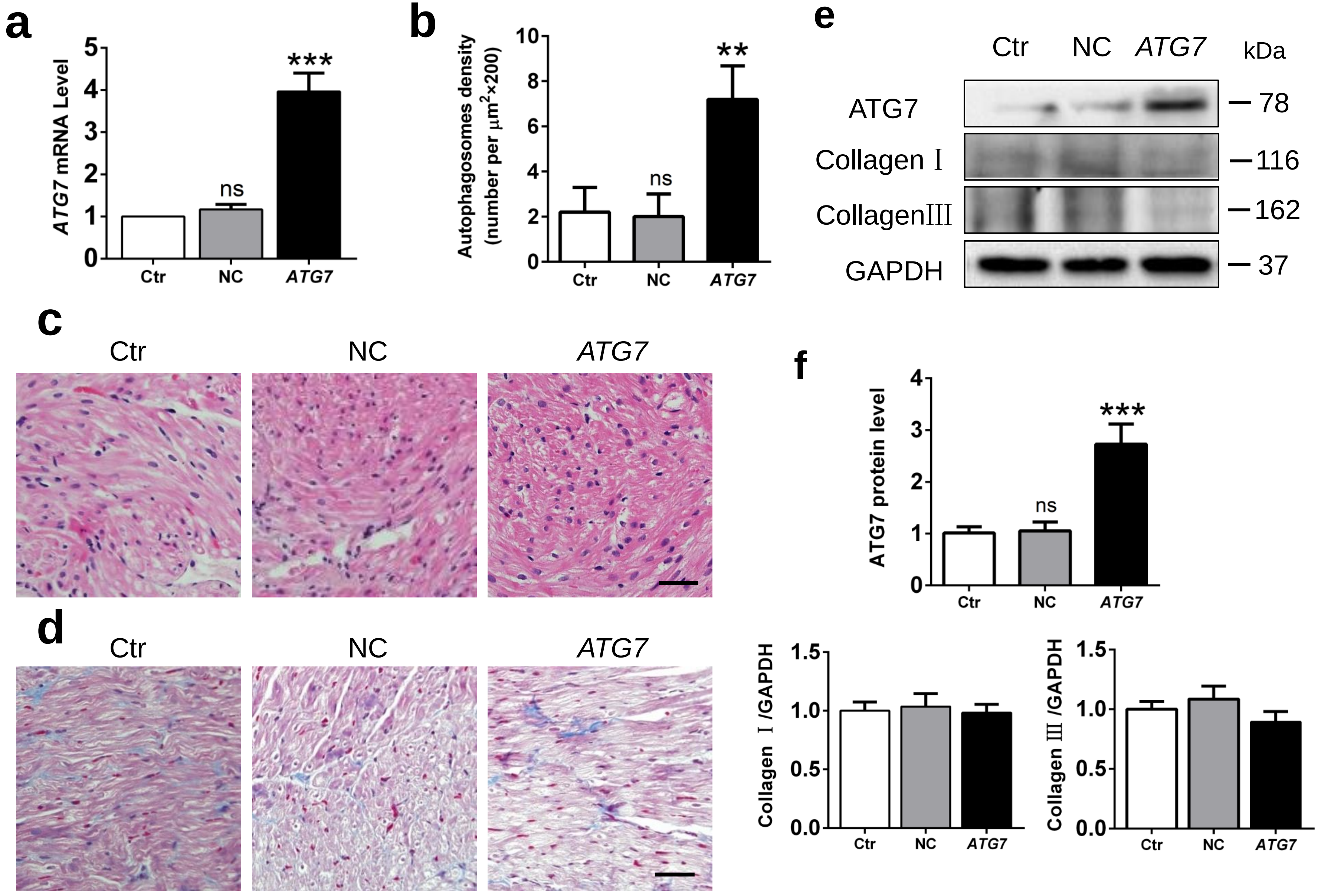

Supplemental Figure 8

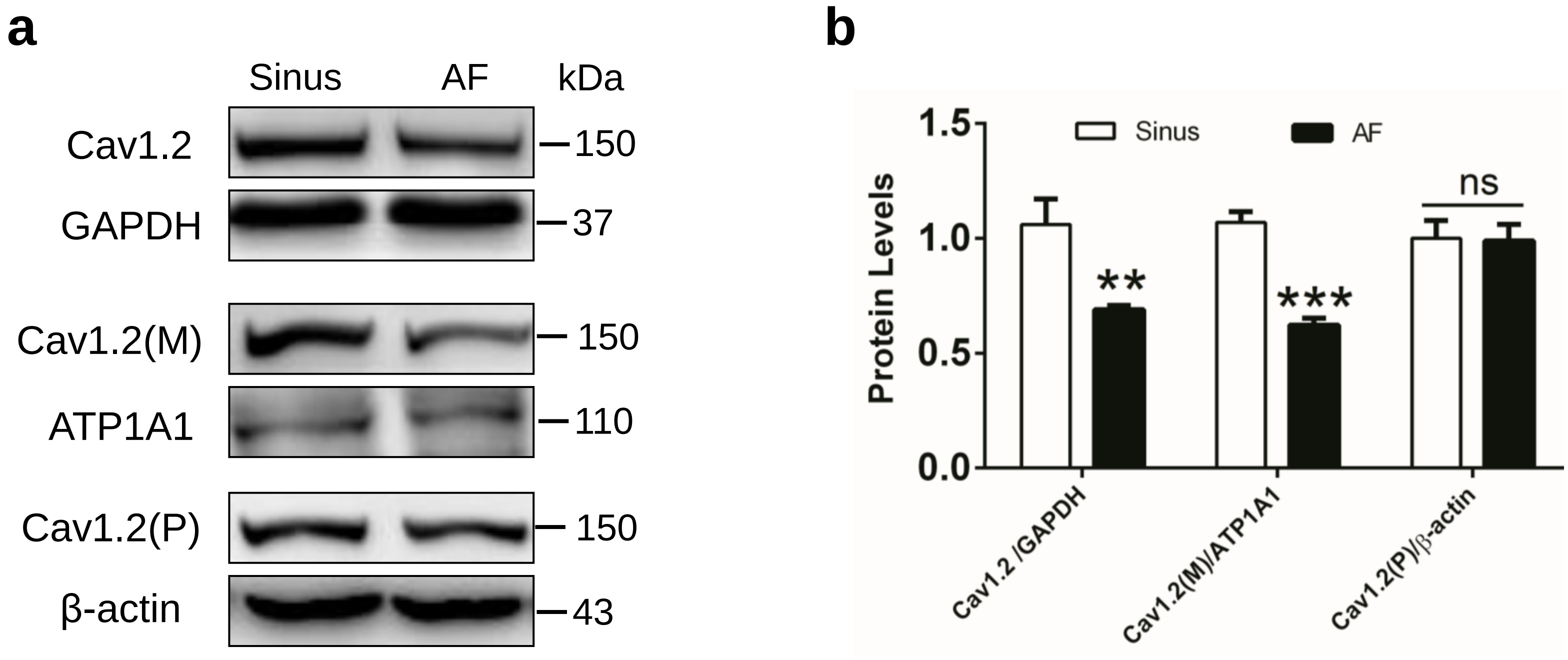

Supplemental Figure 9

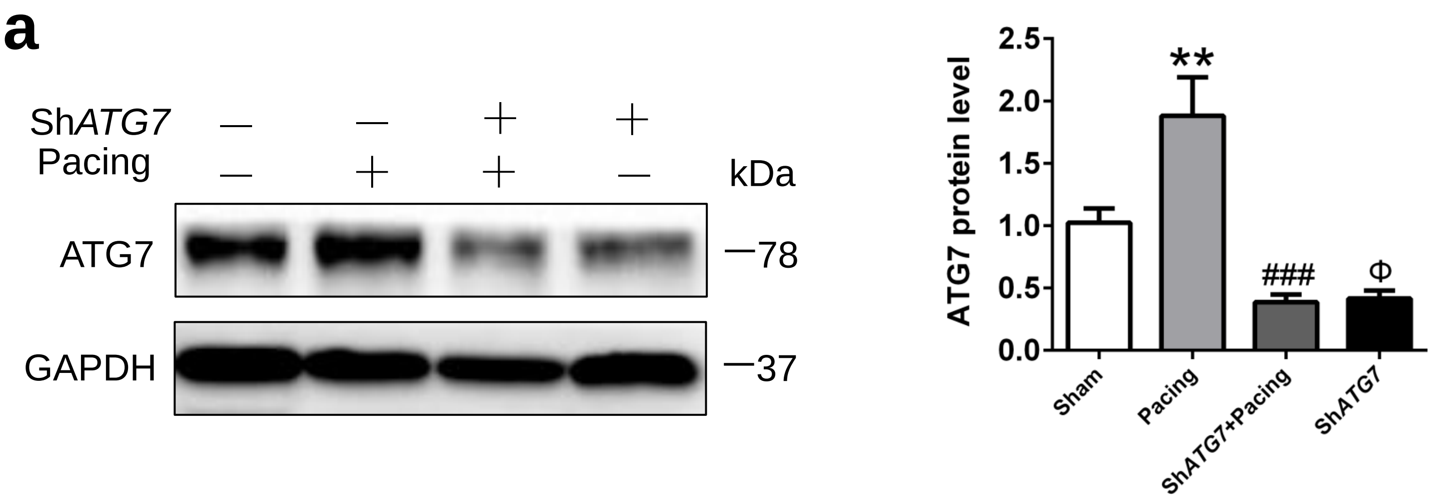

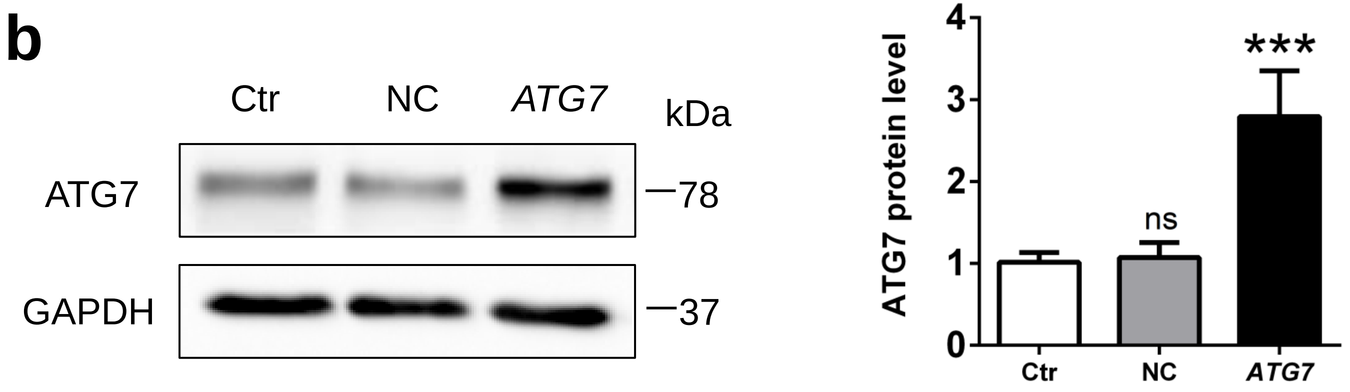

Supplemental Figure 10

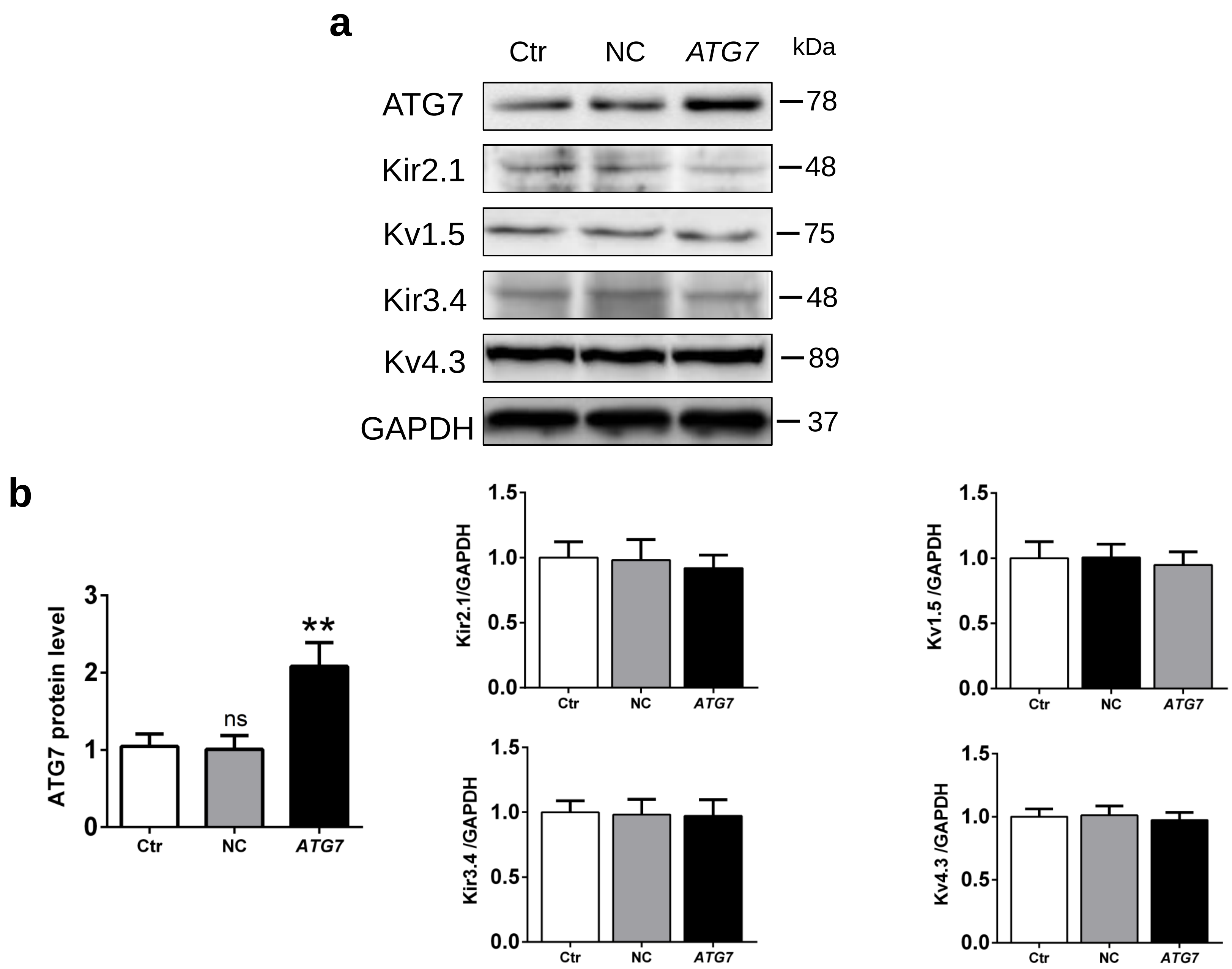

Supplemental Figure 11

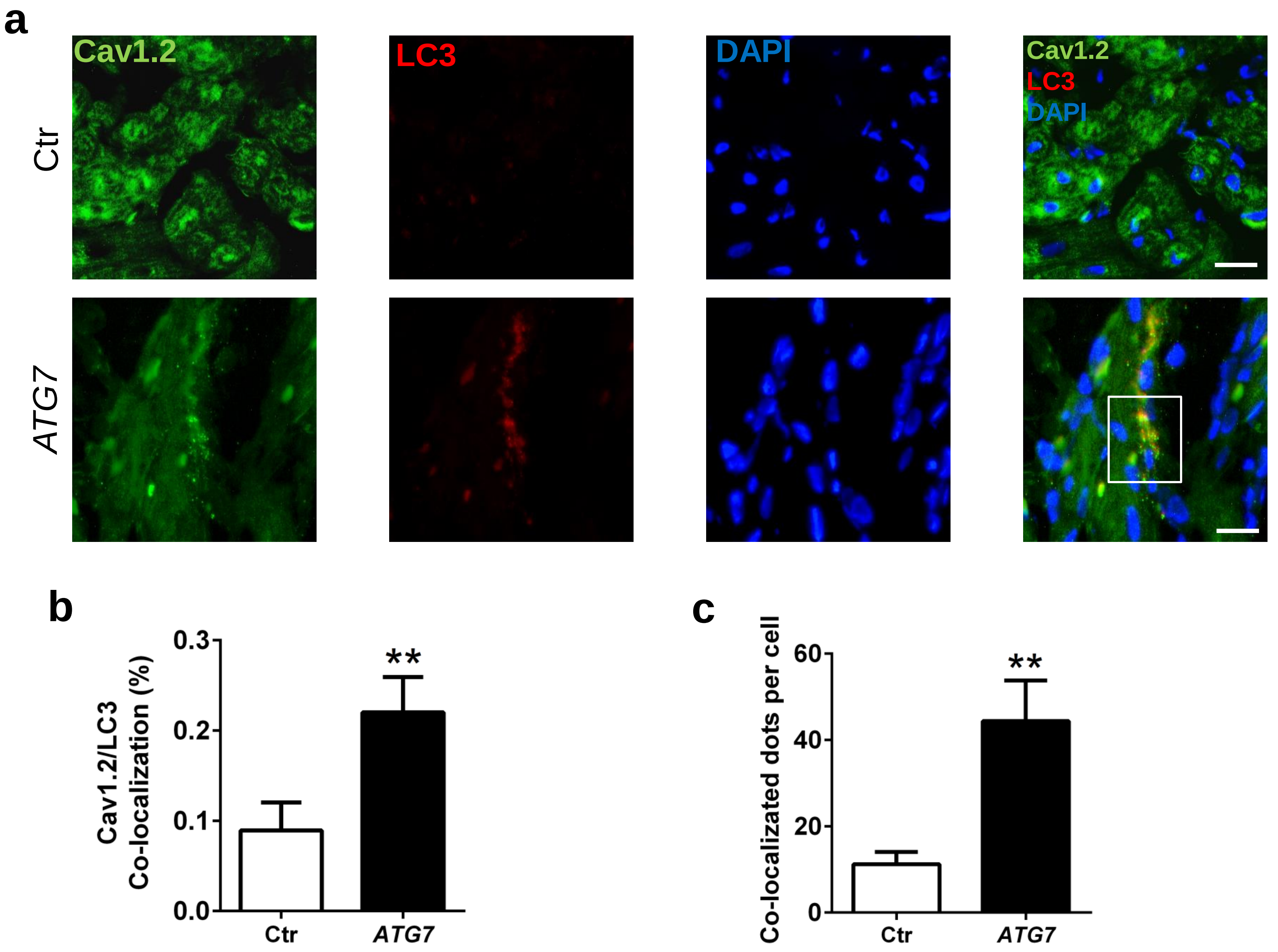

Supplemental Figure 12

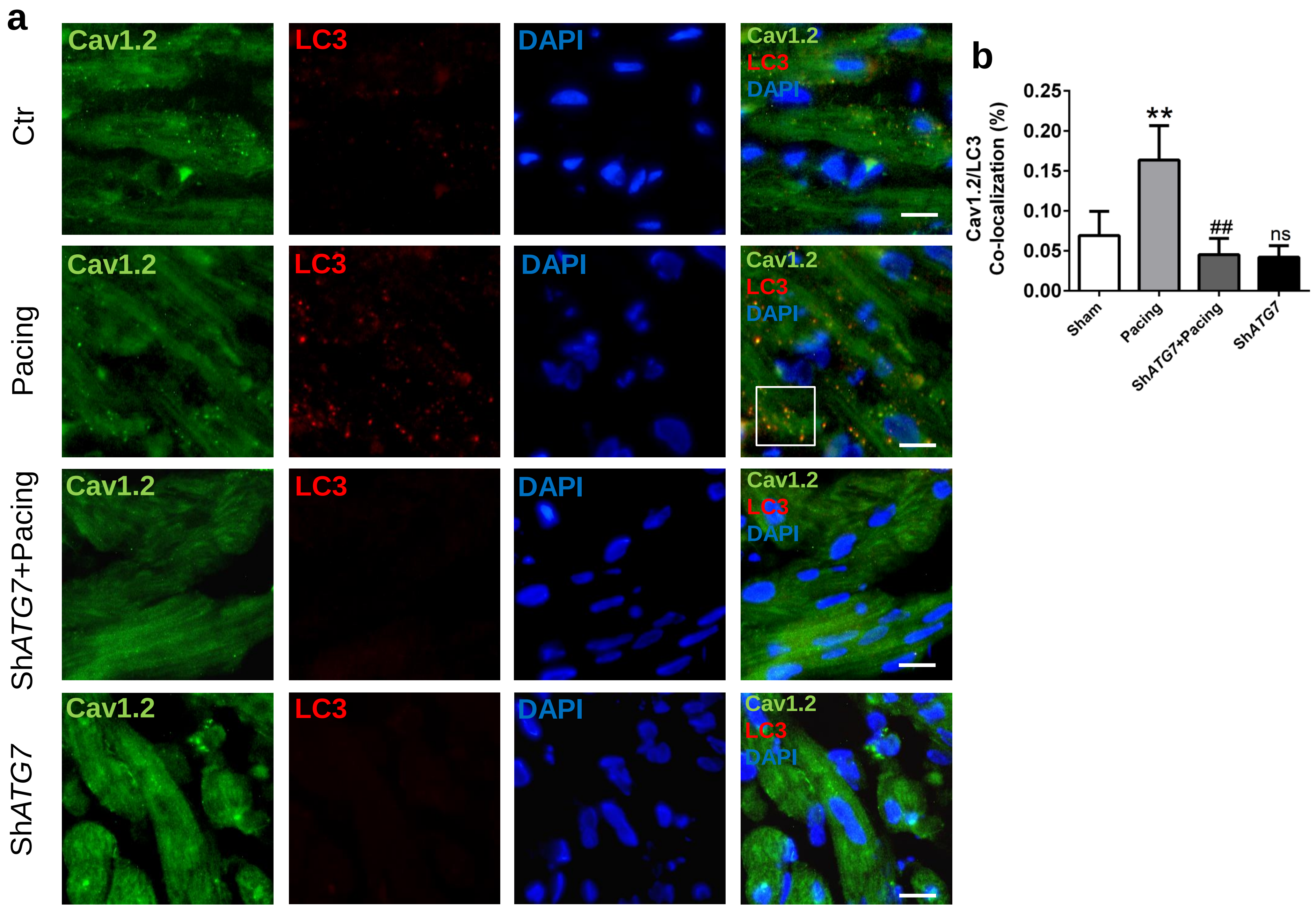

Supplemental Figure 13

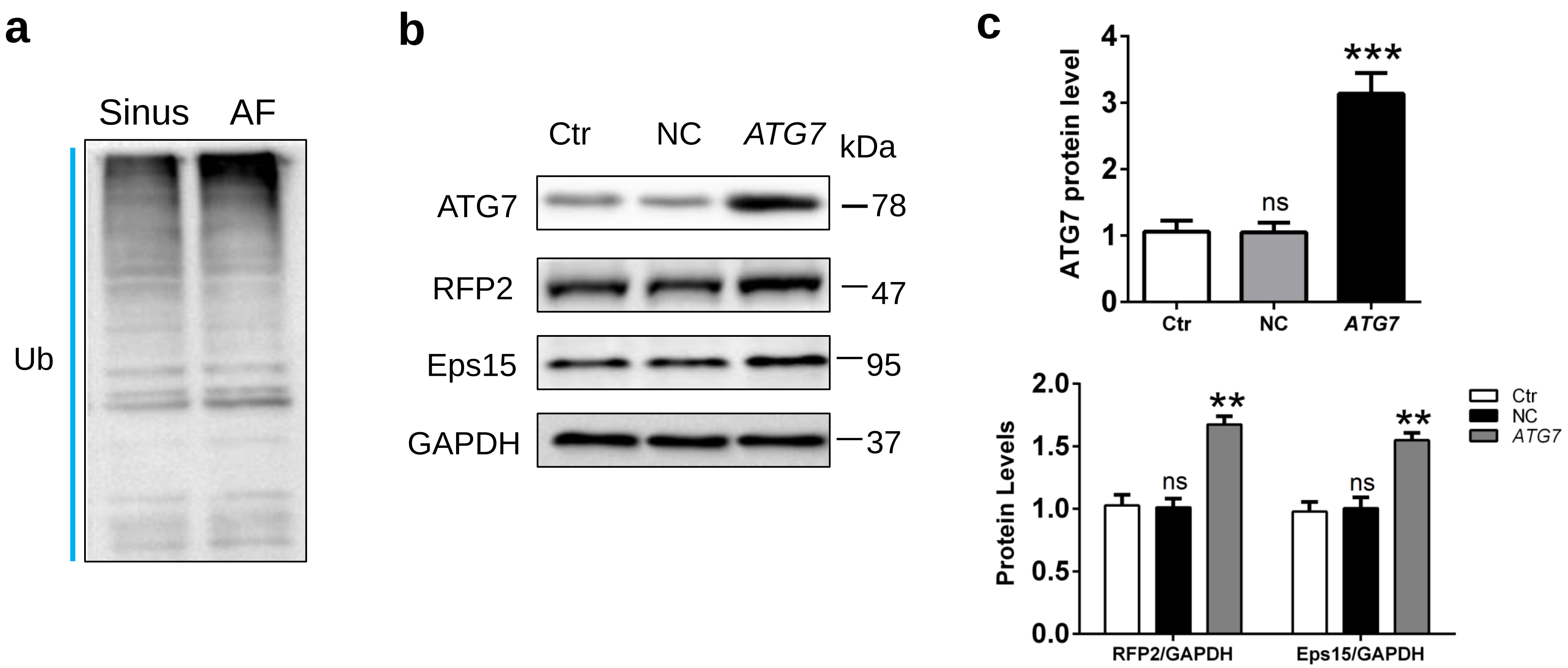

Supplemental Figure 14

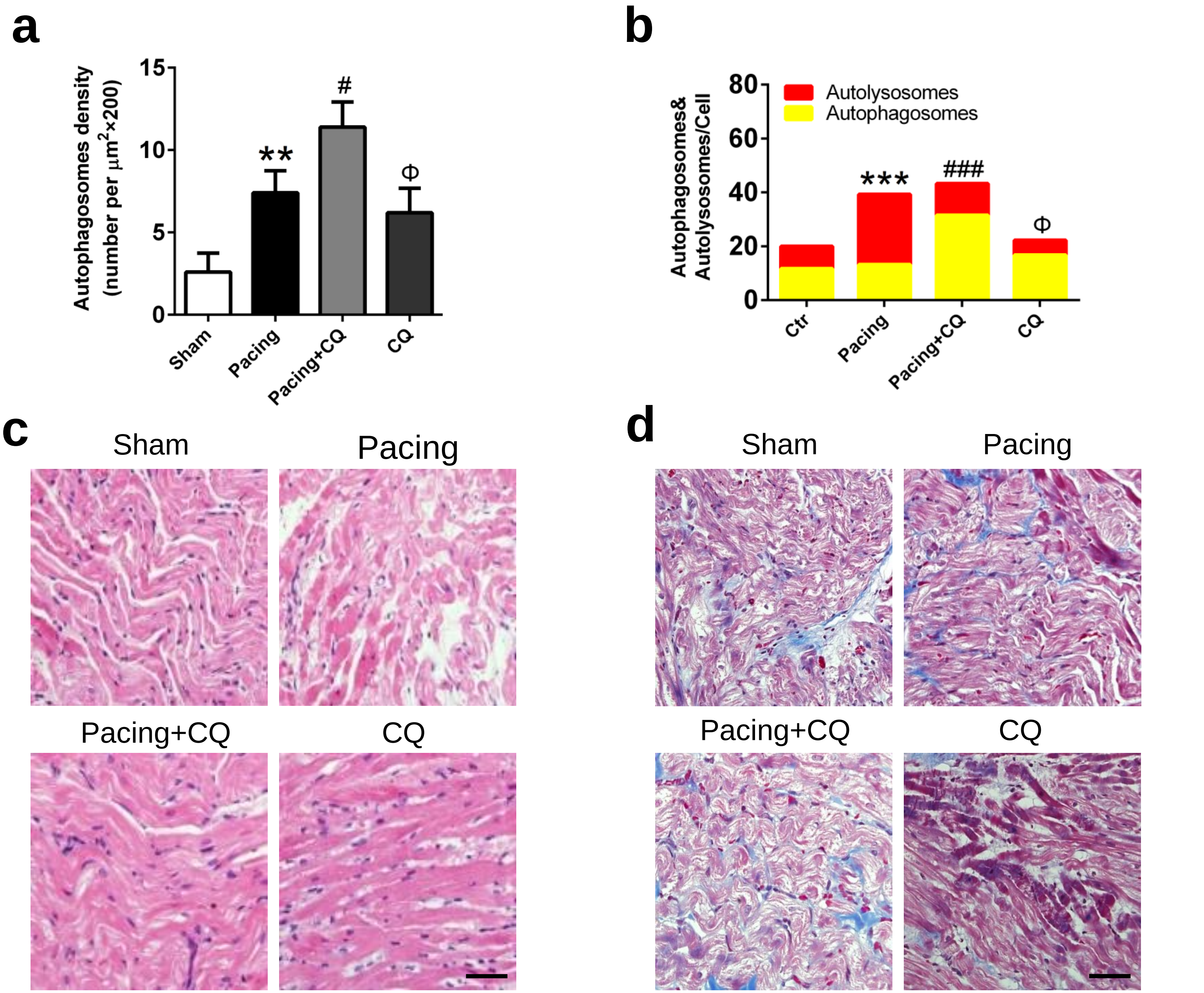

Supplemental Figure 15

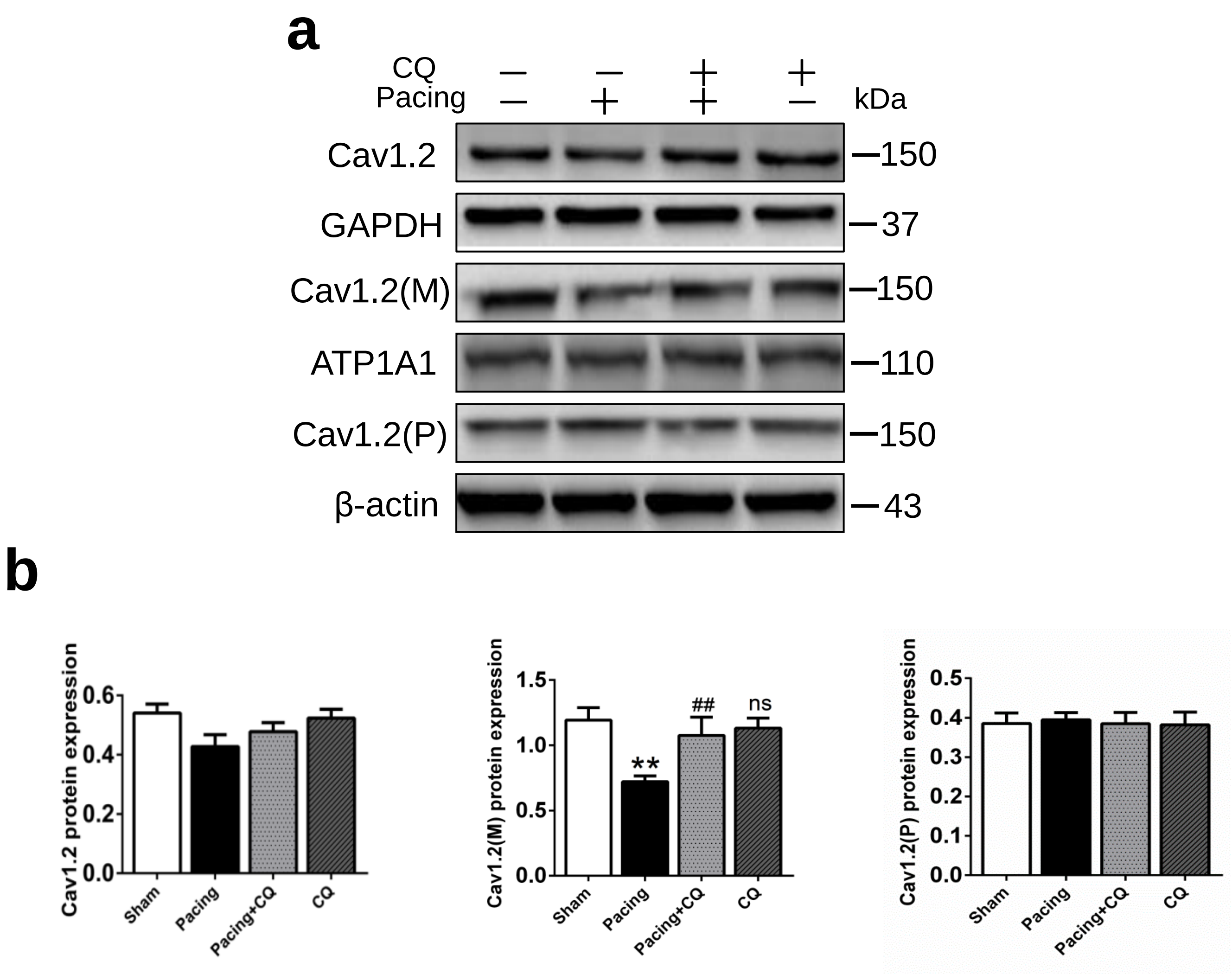

Supplement: Supplementary file 1 — Supplemental Figure [file 41419_2018_860_MOESM1_ESM.pdf]
