# Supplementary material for: Autophagy exacerbates electrical remodeling in atrial fibrillation by ubiquitin-dependent degradation of L-type calcium channel
Source: Cell Death Dis. 2018 Aug 29;9(9):873. doi: 10.1038/s41419-018-0860-y (PMC6115437; doi:10.1038/s41419-018-0860-y)
Supplement: Supplementary file 2 — Supplemental Data [file 41419_2018_860_MOESM2_ESM.docx]

**Supplemental Data**

**
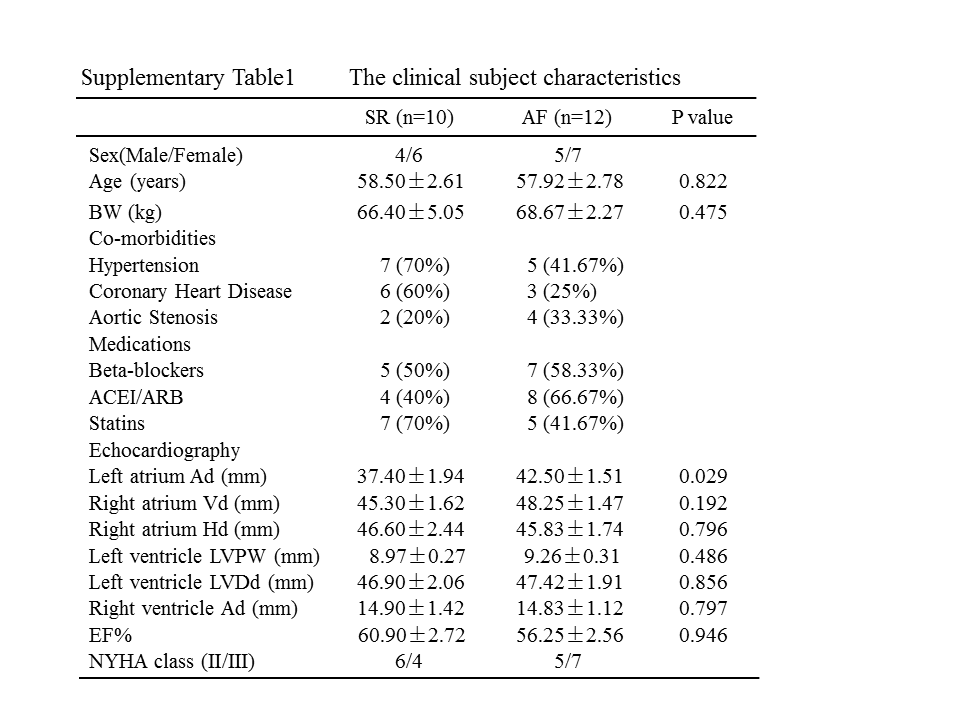
**

**
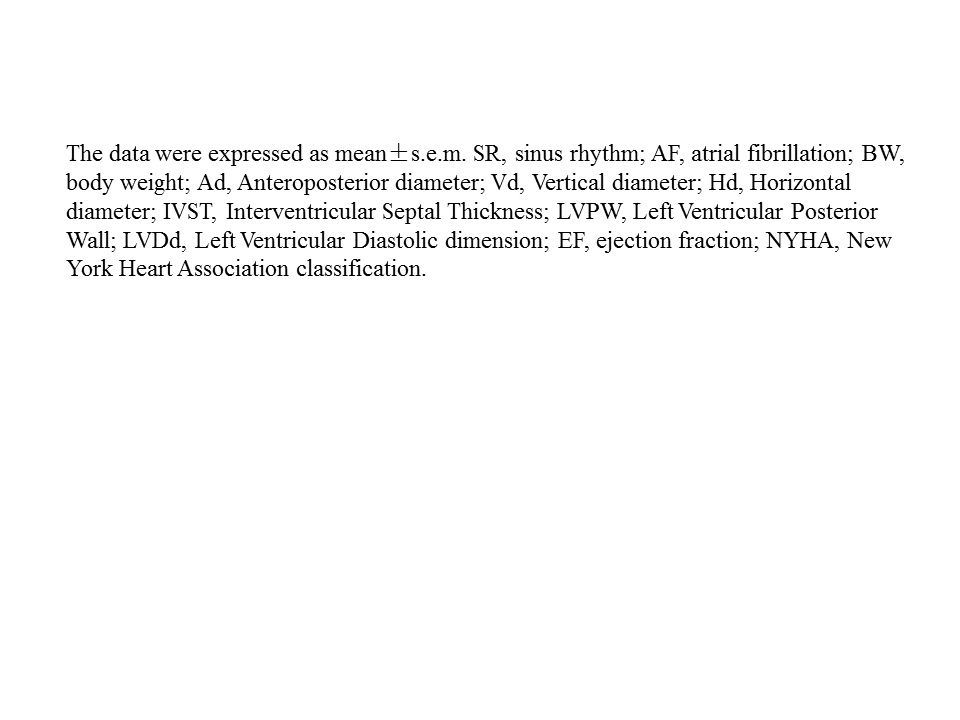
**

**Supplemental Table. 2 Echocardiographic data in rabbits**

**
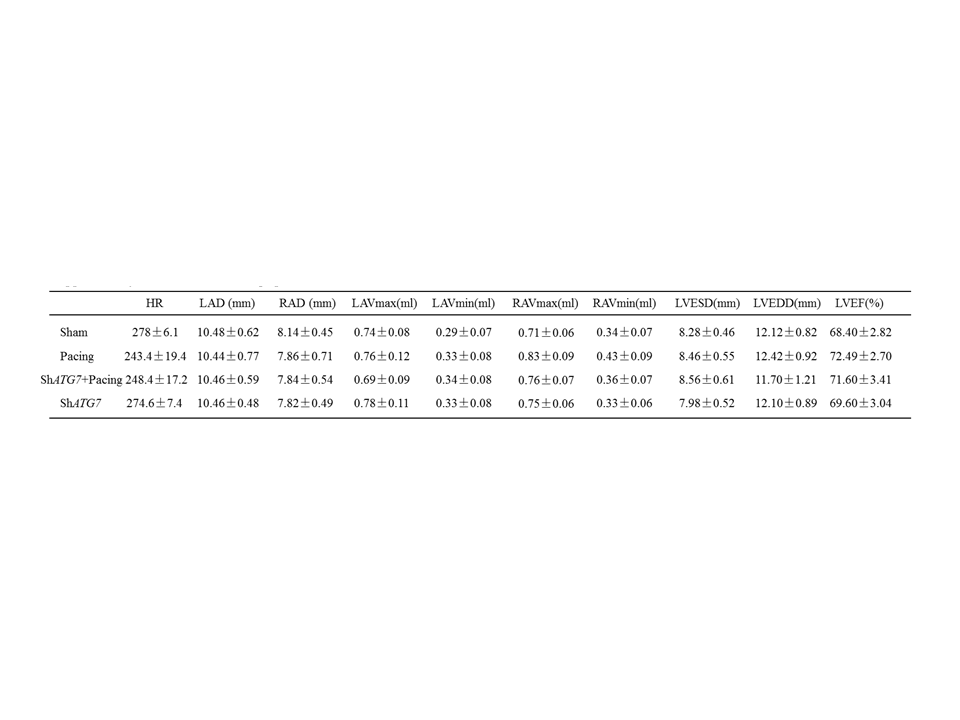
**

**Supplemental Table 3. Echocardiographic data in rabbits**

**
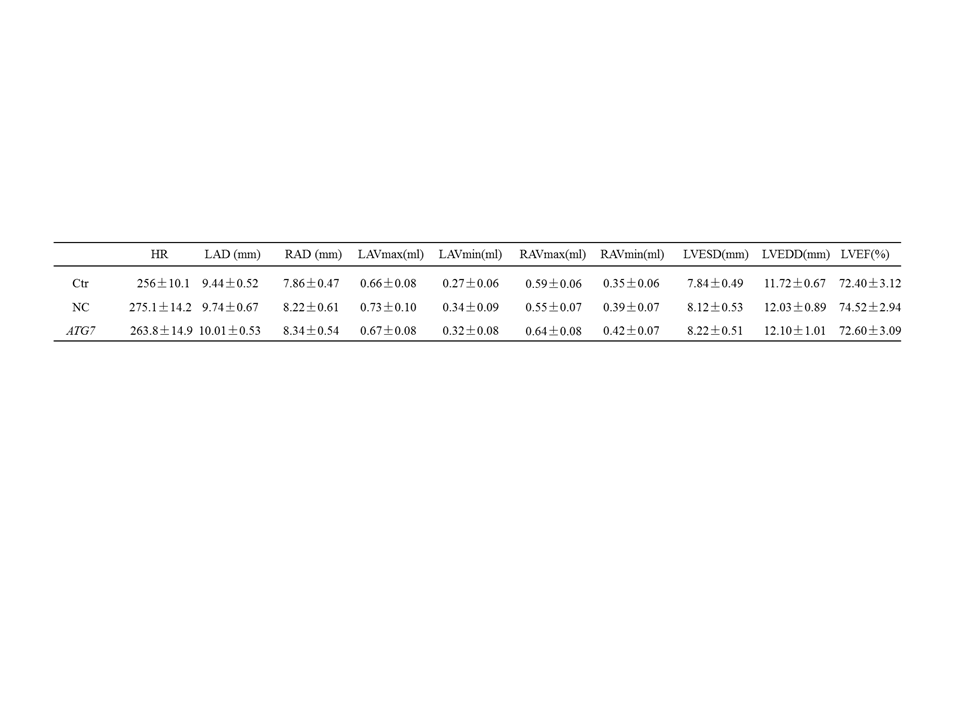
**

**Abbreviations:**

| **AF** | atrial fibrillation |
| --- | --- |
| **AERP** | atrial effective refractory period |
| **APD** | action potential duration |
| **Ca^2+^** | calcium ion |
| **CQ** | chloroquine |
| ***I*_Ca,L_** | L-type calcium channel currents |
| **RAP** | rapid atrial pacing |
| **Ub** | ubiquitin |
| **SR** | sinus rhythm |

**Methods:**

**Western Blot.**

The protein bands were analyzed with Image Lab, and GADPH was used as an internal control. β-actin was used as plasma protein control, and ATP1A1 was used as cellular membrane protein control. LC3B (1:1000), P62 (1:1000) and ATG7 (1:1000) were purchased from Cell Signaling Technologies (Danvers, USA). P62 (1:1000), LAMP2 (1:1000), and Cav1.2 (1:600) were purchased from Abcam (Dallas, USA). Ubiquitin (1:1000) and RFP2 (1:1000) were purchased from Thermo Fisher (CA, USA). Kir2.1 (1:500), Kv1.5 (1:1000), Kir3.4 (1:500) and Kv4.3 (1:1000) were purchased from Bioss (Beijing, China). β-actin (1:1000), ATP1A1 (1:1000), Eps15 (1:1000), CollagenⅠ(1:500) and CollagenⅢ (1:500) were purchased from Proteintech (Wuhan, China). GAPDH (1:1000) was purchased from Kangcheng (Shanghai, China). And the antibody peptide was purchased from Sigma.

**Preparation of cellular and plasma membrane lysates.**

Cellular and plasma membrane lysates were extracted by “Membrane and Cytosol Protein Extraction kit” (Beyotime P0033, China). 100 mg atrial tissue were harvested and cut into small pieces, then added 1 ml mixture of reagent A and PMSF. Then mix pieces by resuspending three times and place on ice for 10 min. Grind the mixture for 30-50 times and draw 2 μl homogenate on the slide. Then observe that there are 70-80% cells destroyed without shiny ring around the nuclei, which indicates the cells are fully squashed. The complex is centrifuged at 700×g for 10 min at 4℃. Draw the supernatant liquid and spin at 14,000×g for 30 min at 4℃, and obtain the plasma protein. Add 200 μl reagent B into the sediment and place on the ice 10 min, then vortex 5 second for 3 times. The complex is centrifuged at 14,000×g for 5 min at 4℃.Then extract the cellular membrane protein.

**Immunohistochemistry.**

The procedure for immunohistochemistry staining has been described previously [1]. The consecutive sections were spliced from the formalin-fixed and paraffin-embedded specimens. Then specimens were dewaxed and incubated with 3% H_2_O_2_ in methanol at 37°C for 10 min to quench the endogenous peroxidase activity. After high-pressure repair for 4 min, the specimens were blocked with 1% bovine fetal serum for 20 min. Then, the sections were incubated with a monoclonal antibody against ATG7 (1:100, CST) overnight at 4℃. Subsequently, the sections were incubated with secondary antibodies (1:200 dilution; ZSGB-BIO Inc., Beijing, China) and developed for color with DAB peroxidase color development kits. Finally, the specimens were counterstained with hematoxylin. The tissues were analyzed under a microscope (100×) and cells with the presence of dark reddish-brown chromogen were identified as positive.

**Sequences of qRT-PCR primers.**

ATG1 Human: Forward primer (5’-3’) CTGGTCCTCTTGCTTCCGTC

ATG1 Human: Reverse primer (5’-3’) CACCAGCCCAACAATTCCA

ATG3 Human: Forward primer (5’-3’) GAGCAACGGCAGCCTTTAAC

ATG3 Human: Reverse primer (5’-3’) CGTTAACAGCCATTTTGCCAC

ATG4 Human: Forward primer (5’-3’) GTGGAATTGGCCCAGGATGA

ATG4 Human: Reverse primer (5’-3’) AGCATACATCCCCAACCAGC

ATG5 Human: Forward primer (5’-3’) TGCAGATGCACAGTTGCACA

ATG5 Human: Reverse primer (5’-3’) CCACTGCAGAGGTGTTTCCA

ATG6 Human: Forward primer (5’-3’) CTCCCGAGGTGAAGAGCATC

ATG6 Human: Reverse primer (5’-3’) GGGGGATGAATCTGCGAGAG

ATG7 Human: Forward primer (5’-3’) CAGCAGTGACGATCGGATGA

ATG7 Human: Reverse primer (5’-3’) GCTCATGTCCCAGATTTTGGA

ATG8 Human: Forward primer (5’-3’) ATTGACACACTGGCGGACTT

ATG8 Human: Reverse primer (5’-3’) AATCCCCTTGGCTCACGTTT

ATG10 Human: Forward primer (5’-3’) GAAGCCGGAAGGGGTTCAA

ATG10 Human: Reverse primer (5’-3’) CAGGGAGTCAAAAACTGCCC

ATG12 Human: Forward primer (5’-3’) AAGTGGGCAGTAGAGCGAAC

ATG12 Human: Reverse primer (5’-3’) CACGCCTGAGACTTGCAGTA

ATG13 Human: Forward primer (5’-3’) TGGCCTCTTAGGGGCTGTAT

ATG13 Human: Reverse primer (5’-3’) AAATCCCCCAGATGGCTTGG

ATG7 Rabbit: Forward primer (5’-3’) CGACAAATGCACCGCTTGTT

ATG7 Rabbit: Reverse primer (5’-3’) ATGAAACGCACAGCACCTCT

ATG7 Mouse: Forward primer (5’-3’) GTCACAGCCCTGCCATACTT

ATG7 Mouse: Reverse primer (5’-3’) CATCCAGGGTGCTGGGTTAG

GAPDH Human: Forward primer (5’-3’) CCTCTGACTTCAACAGCGACAC

GAPDH Human: Reverse primer (5’-3’) TGGTCCAGGGGTCTTACTCC

GAPDH Rabbit: Forward primer (5’-3’) CGAGACACGATGGTGAAGGT

GAPDH Rabbit: Reverse primer (5’-3’) CCAGCATCACCCCACTTGAT

GAPDH Mouse: Forward primer (5’-3’) TGGAGTCTACTGGCGTCTT

GAPDH Mouse: Reverse primer (5’-3’) TGTCATATTTCTCGTGGTTCA

**Reference**

1. Liu G, Hou T, Yuan Y, et al. Fenofibrate inhibits atrial metabolic remodeling in atrial fibrillation through PPAR-α/sirtuin1/PGC-1α pathway. *Br J Pharmacol*. 2016;173:1095-109.

**Supplemental figure legends**

**Figure S1 ATG related mRNA and protein level in AF** (a) Quantitation of autophagosomes in atrial tissues of patients. (b) Quantitative RT-PCR (qRT-PCR) analysis of *ATG1*, *ATG3*, *ATG4*, *ATG5*, *ATG6*, *ATG7*, *ATG8*, *ATG12* and *ATG13* mRNA expression in the atrial tissues from patients (n=10 for Sinus and n=12 for AF). ATG7 mRNA levels increased in atrial tissues of AF patients. (c) Immunoblots of ATG5 and BECN1 protein in the atrial tissues of sinus and AF patients. (d) The images of negative and positive controls for ATG7 immunochemistry (original magnification, 100×). Scale bar: 50 μm.

**Figure S2 The original bands of Figure 1b and the negative and positive controls in western blot analysis** (a) The original bands of LC3B, p62, LAMP2, ATG7 and GAPDH in atrial tissues of patients. (b) The original bands of LC3B, p62, LAMP2, ATG7 and GAPDH with antibody peptide as negative control, human pancreatic tissues as positive control and normal atrial tissues of patients. PC: positive control, NC: negative control, S1 and S2: sinus patients.

**Figure S3 The original bands of Figure 1e in western blot analysis** The original bands of LC3B, p62, LAMP2, ATG7 and GAPDH in atrial tissues of pacing rabbits.

**Figure S4 ATG related mRNA and protein level in AF** (a) The expression of *ATG7* mRNA was analyzed by qRT-PCR in atrial tissues of rapid pacing rabbits. ^**^*P＜*0.01 sham vs. Pacing 1 day; ^###^*P＜*0.001 sham vs. Pacing 7 days; ^φφφ^*P＜*0.001 sham vs. Pacing 14 days. (b and c) Immunoblot results showed expressions of ATG5 and BECN1 protein from atrial rapid pacing rabbit model (n=5 for sham group, n=5 for pacing 1day, n=4 for 7days, and n=5 for AF 14days).

**Figure S5 *ATG7* knockdown did not affect atrial fibrosis level** (a) Verification of *ATG7* knockdown by qRT-PCR in rabbit atrial tissues. ns Ctr vs. NC, ^***^*P＜*0.001 Sh*ATG7-1* vs. Ctr, ^###^*P＜*0.001 Sh*ATG7-2* vs. Ctr, ^φφφ^*P＜*0.001 Sh*ATG7-3* vs. Ctr. (b) *ATG7* mRNA increased in pacing rabbit atria and decreased in Sh*ATG7* group. ^***^*P＜*0.001 sham vs. Pacing; ^###^*P＜*0.001 Pacing vs. Sh*ATG7*+Pacing; ^φφφ^*P＜*0.001 sham vs. Sh*ATG7*. (c) H&E stained sections of atria subjected to pacing and Sh*ATG7* (original magnification, 100×). Scale bar: 50 μm. (d) Quantitation of autophagosomes and autolysosmes in atrial tissues of rabbits. ^**^*P＜*0.01 Pacing vs. Sham, ^##^*P＜*0.01 Pacing vs. Pacing+Sh*ATG7*. (e) Masson stained sections of atria subjected to pacing and Sh*ATG7* (original magnification, 100×). Scale bar: 50 μm. (f) Western blot results showed no changes of CollagenⅠ and CollagenⅢ protein in these four groups.

**Figure S6 *ATG7* SiRNA inhibited autophagy process in tachypaced HL-1 cells** (a) ATG7 mRNA reduced by siRNA in HL-1 cells. ^***^*P＜*0.001 in one-way analysis of variance. (b) Electron microscopy analysis in HL-1 cells treated with pacing or knockdown *ATG7* in HL-1 cell (original magnification, 20,000×). Ctr: control group; Pacing: 5Hz tachypacing group; Si*ATG*7+Pacing: knockdown *ATG7* with tachypacing group; Si*ATG*7: knockdown *ATG7* group. Scale bar: 500 nm. (c and d) Representative images of mRFP-GFP-LC3 showed free red dots in tachypacing cells, and yellow dots in tachypacing+Si*ATG7* group. ^***^*P＜*0.001 Ctr vs. Pacing, ^##^*P＜*0.01 Si*ATG*7+Pacing vs. Pacing, ns Ctr vs. Si*ATG*7.

**Figure S7 The fibrosis level in overexpressing *ATG7* rabbits** (a) ATG7 mRNA was significantly increased in *ATG7* rabbits. ns Ctr vs. NC, ^***^*P＜*0.001 Ctr vs. *ATG7*. (b) The number of autophagosomes and autolysosomes was increased in atrial tissues of *ATG7* overexpression rabbits. (c and d) The images of H&E and Masson showed morphology and fibrosis of rabbit atria (original magnification, 100×). Scale bar: 50 μm. (e and f) The CollagenⅠand Collagen Ⅲ protein levels sustained unchanged by immunobloting. Immunoblot results showed the expression of ATG7 protein from atrial *ATG7* overexpression rabbit model. ns Ctr vs. NC; ^***^*P＜*0.001 Ctr vs. *ATG7.*

**Figure S8 Cav1.2 protein expression in AF patients** (a and b) Total, cellular membrane and plasma Cav1.2 proteins in atrial tissues of Sinus and AF patients were tested by immunoblots. ^**^*P＜*0.01; ^***^*P＜*0.001; ns means no statistical significance in Student’s *t*-test analysis AF group compared with Sinus group.

**Figure S9 ATG7 protein level in atrial tissues of rabbits** (a) The expression of ATG7 protein was analyzed in atrial tissues of rapid pacing and Sh*ATG7* rabbits. ^**^*P＜*0.01 Sham vs. Pacing; ^###^*P＜*0.001 Pacing vs. Sh*ATG7*+Pacing; ^φ^*P＜*0.05 Sham vs. Sh*ATG7*. (b) Immunoblot results showed the expression of ATG7 protein from atrial *ATG7* overexpression rabbit model. ns Ctr vs. NC; ^***^*P＜*0.001 Ctr vs. *ATG7.*

**Figure S10 Atrial ion channel expression in overexpressing *ATG7* rabbits** (a and b) Immunoblot of Kir2.1, Kv1.5, Kir3.4 and Kv4.3 protein in atrial tissues of *ATG*7 overexpression rabbits. There were no changes in three groups. Immunoblot results showed the expression of ATG7 protein from atrial *ATG7* overexpression rabbit model. ns Ctr vs. NC; ^**^*P＜*0.01 Ctr vs. *ATG7.*

**Figure S11 The co-localization of LC3B and Cav1.2 in *ATG*7 overexpression rabbits** (a and b) Immunofluorescence showed the co-localization between LC3B and Cav1.2 was increased in atrial tissues of *ATG7* overexpression rabbits. Scale bar: 25μm. (c) The quantitation of co-localization between LC3B and Cav1.2 in atrial cardiomyocytes isolated from *ATG7* overexpression rabbits. ^**^*P＜*0.01 Ctr vs. *ATG7.*

**Figure S12 The co-localization of LC3B and Cav1.2 was decreased by *ATG*7 knockdown** (a and b) Immunofluorescence showed the co-localization between LC3B and Cav1.2 was increased in atrial tissues of pacing rabbits, which was blocked by Sh*ATG7*. Scale bar: 25μm. ^**^*P＜*0.01 Pacing vs. Sham; ^##^*P＜*0.01 Sh*ATG7*+Pacing vs. Pacing.

**Figure S13 The ubiquitin signal in AF** (a) The ubiquitin expression in atrial tissues of sinus and AF patients. (b and c) Immunoblots of RFP2 and Eps15 in atrial tissues of *ATG7* overexpression rabbits. ns Ctr vs. NC, ^**^*P＜*0.01 Ctr vs. *ATG7.* Immunoblot results showed the expression of ATG7 protein from atrial *ATG7* overexpression rabbit model. ns Ctr vs. NC; ^***^*P＜*0.001 Ctr vs. *ATG7.*

**Figure S14 CQ did not affect atrial structural damage** (a) Quantitation of autophagosomes and autolysosmes in atrial tissues of rabbits. ^**^*P＜*0.01 Pacing vs. Sham, ^#^*P＜*0.05 Pacing vs. Pacing+CQ. (b) Quantitation of autophagosomes and autolysosmes in HL-1 cells by mRFP-GFP-LC3 system. ^***^*P＜*0.001 Pacing vs. Sham, ^###^*P＜*0.001 Pacing vs. Pacing+CQ. (c and d) H&E and Masson stained sections of atria subjected to pacing and CQ (original magnification, 100×). Scale bar: 50 μm.

**Figure S15 CQ inhibited autophagy-mediated degradation of Cav1.2 protein** (a　and b) Total, cellular membrane and plasma Cav1.2 proteins in atrial tissues of pacing rabbits in absence or presence of CQ were tested by immunoblots. ^**^*P＜*0.01; Sham vs. Pacing. ^#^*P＜*0.05; ^##^*P＜*0.01; ^###^*P＜*0.001 Pacing vs. Pacing+CQ. ns Ctr vs. CQ.
